# Visualization of long-term Mg^2+^ dynamics in apoptotic cells using a novel targetable fluorescent probe†
†Electronic supplementary information (ESI) available. See DOI: 10.1039/c7sc03954a


**DOI:** 10.1039/c7sc03954a

**Published:** 2017-10-20

**Authors:** Yusuke Matsui, Yosuke Funato, Hiromi Imamura, Hiroaki Miki, Shin Mizukami, Kazuya Kikuchi

**Affiliations:** a Department of Material and Life Science , Graduate School of Engineering , Osaka University , Suita , Osaka 565-0871 , Japan . Email: kkikuchi@mls.eng.osaka-u.ac.jp; b Department of Cellular Regulation , Research Institute for Microbial Diseases , Osaka University , Suita , Osaka 565-0871 , Japan; c Graduate School of Biostudies , Kyoto University , Kyoto 606-8501 , Japan; d Institute of Multidisciplinary Research for Advanced Materials , Tohoku University , Sendai , Miyagi 980-8577 , Japan . Email: shin.mizukami@m.tohoku.ac.jp; e Immunology Frontier Research Center , Osaka University , Suita , Osaka 565-0871 , Japan

## Abstract

Mg^2+^ plays important roles in many physiological processes. However, the underlying molecular mechanisms, especially in the apoptotic pathway, remain unclear due to the diffusion of Mg^2+^ probes, which hinders long-term imaging in specific organelles. We developed an immobilized Mg^2+^ probe, MGH, which is covalently conjugated with the HaloTag protein in various organelles. HaloTag-coupled MGH enabled long-term imaging of intracellular local Mg^2+^ dynamics for 24 h. To exploit this remarkable property, MGH was applied to the investigation of intracellular Mg^2+^ dynamics during apoptosis. Time-lapse imaging revealed an increase in the Mg^2+^ concentration after apoptotic cell shrinkage. Combined imaging analyses of intracellular Mg^2+^ and ATP concentrations strongly suggested that this Mg^2+^ concentration increase was caused by the dissociation of Mg^2+^ from ATP, along with a decrease in the intracellular ATP concentration. Thus, this protein-coupled Mg^2+^ probe could be a new chemical tool to elucidate intracellular Mg^2+^ dynamics with high spatiotemporal resolution.

## Abstract 2

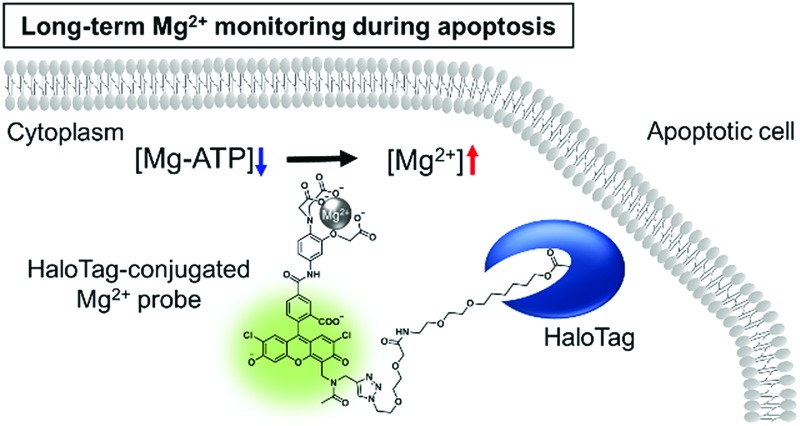
Long-term Mg^2+^ imaging during apoptosis using a HaloTag-coupled Mg^2+^ probe demonstrated a Mg^2+^ concentration increase caused by dissociation of Mg^2+^ from ATP.

## Introduction

Mg^2+^ is an essential divalent cation in cells, and the overall intracellular Mg^2+^ concentration ranges between 17 and 20 mM. However, the intracellular free Mg^2+^ concentration ([Mg^2+^]_i_) is maintained between 0.5 and 1 mM, because most Mg^2+^ forms complexes with nucleotides, such as ATP or DNA.^1^,^2^ Mg^2+^ plays vital roles in a variety of physiological processes such as regulation of enzyme activities, stabilization of nucleotides, and cell proliferation.^2^–^4^ It is understood that intracellular Mg^2+^ homeostasis is tightly regulated by Mg^2+^ transporters and Mg^2+^ channels and buffered by intracellular ATP.^1^ In addition, Mg^2+^ plays a role as a secondary messenger in T cells.^5^ Abnormal Mg^2+^ homeostasis is involved in several disorders including diabetes, hypertension, Parkinson’s disease, and cancer.^6^,^7^

Despite Mg^2+^ being obviously important for regulating cellular functions, regulation of the underlying molecular mechanisms of [Mg^2+^]_i_ still remains unclear.^8^ One phenomenon for which Mg^2+^ dynamics are unclear is apoptosis. Apoptosis is a programmed cell death process occurring over several hours to eliminate damaged cells. While Ca^2+^ plays very important roles in regulating the apoptotic process,^9^ changes in [Mg^2+^]_i_ have also been observed in apoptotic cells, for example hepatocytes treated with glycodeoxycholate^10^ and B lymphocytes treated with Fas ligand.^11^ However, the intracellular Mg^2+^ dynamics during apoptosis have not been continuously visualized due to the lack of suitable analytical tools. To date, commercially available Mg^2+^ fluorescent probes, such as magnesium green and Mag-Fura-2, have often been utilized to detect free Mg^2+^ in cells. However, these probes tend to quickly diffuse over the entire cytoplasm and leak out of the cells *via* anion transporters.^12^,^13^ These unfavorable properties have made it impossible to image local Mg^2+^ concentration changes over long time periods, resulting in insufficient information regarding the intracellular Mg^2+^ dynamics in apoptotic cells.

To improve the spatial and temporal resolution of Mg^2+^ imaging, various Mg^2+^ probes have been developed. Recently, genetically encoded fluorescent protein-based Mg^2+^ sensors, MagFRET^14^ and MagIC,^15^ have been reported. These probes were easily targeted to various intracellular compartments by adding a localization signal peptide, and are likely to be capable of long-term detection. However, the application of these probes is limited, probably due to their low sensitivity in living cells^14^ and/or pH sensitivity under physiological conditions.^15^ Therefore, ideal protein-based Mg^2+^ sensors have not yet been developed.

Therefore, small molecule-based Mg^2+^ probes with improved fluorescence properties and intracellular behaviors have been developed for the exploration of intracellular Mg^2+^ dynamics. A chemical Mg^2+^ sensor functionalized with a lipophilic cationic alkylphosphonium group, Mag-mito, showed targetability to mitochondria.^16^ Mag-mito detected changes in the free Mg^2+^ concentration in mitochondria by calculating the ratio of fluorescence signals obtained using two different excitation wavelengths. Mag-mito successfully detected an increase in free Mg^2+^ in mitochondria in the early stages of staurosporine-induced apoptosis. However, this probe diffused from the mitochondria to the cytosol after 40 min of the compound treatment due to the depolarization of the mitochondrial membrane during apoptosis.^16^ It is likely that such noncovalent targetable probes diffuse away from the target site during long-term experiments. Furthermore, for monitoring Mg^2+^ concentration in various other organelles, novel fluorescent probes with different targeting moieties must be developed.

Therefore, a versatile method is to use a protein labeling system, with a pairing of a genetically encoded tag and its specific ligand. So far, several targetable fluorescent sensors have been developed on the basis of protein labeling systems, such as SNAP-tag and HaloTag.^17^–^25^ However, genetically encoded tag-based targeting of Mg^2+^ probes has rarely been reported. One example is KMG-104-AsH,^26^ which was developed based on the highly selective Mg^2+^ sensors in the KMG series.^27^–^29^ KMG-104-AsH can be anchored to a tetracysteine peptide tag (TC-tag), which is genetically expressed in specific organelles, enabling the detection of local Mg^2+^ concentration changes. However, long-term imaging of more than 4 h has not been carried out, probably owing to the cellular toxicity of an additive (ethanedithiol) that is necessary to reduce nonspecific binding.^30^ For the above-mentioned reasons, novel targetable fluorescent probes for long-term monitoring of local Mg^2+^ dynamics in living cells have been pursued.

To address these issues, we developed a tag protein-conjugatable Mg^2+^ probe that can be localized in specific organelles for imaging local Mg^2+^ dynamics in living cells. Through covalent anchoring of the Mg^2+^ probe to a tag protein expressed in various cellular compartments, intracellular diffusion and extracellular leakage of the Mg^2+^ probe were suppressed. This enabled not only the localization of the Mg^2+^ probe, but also long-term imaging of intracellular Mg^2+^ dynamics. This approach provided the first long-term imaging of [Mg^2+^]_i_ during apoptosis.

## Results

### Design and synthesis of the Mg^2+^ probe with the HaloTag ligand

**Fig. 1:**
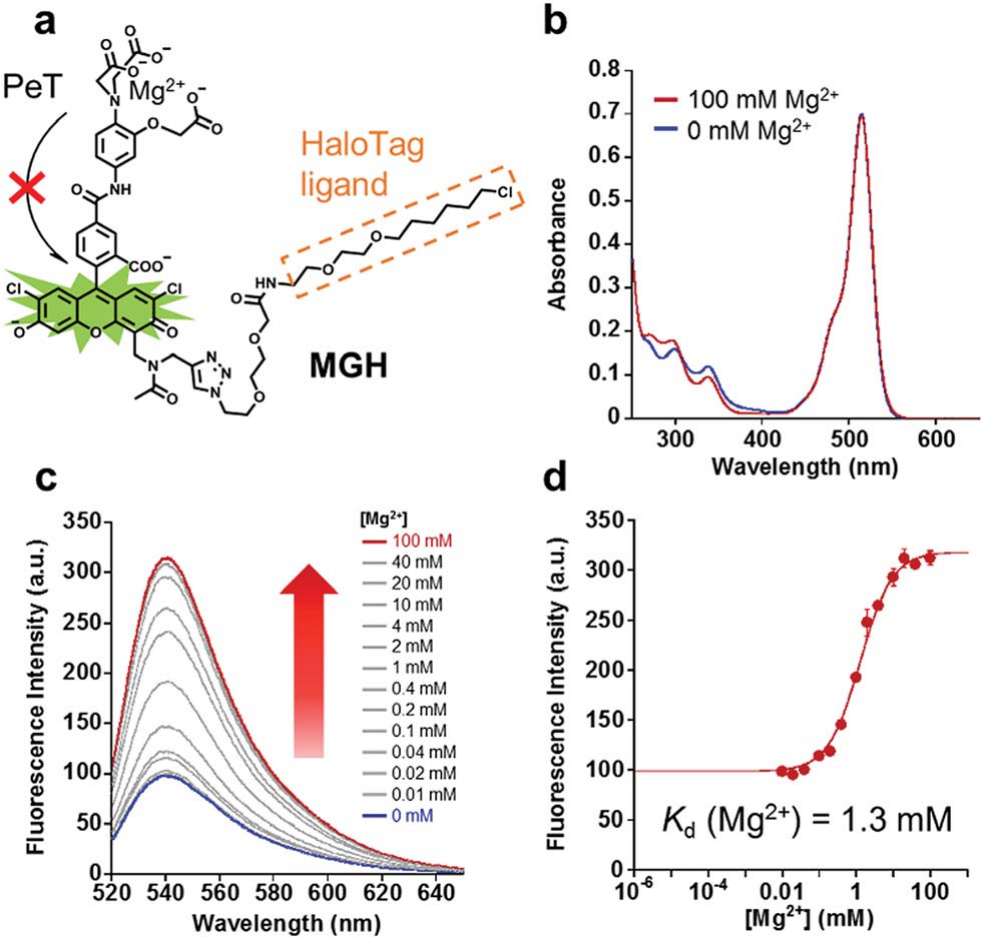
Structure and spectroscopic properties of MGH. (a) An overview of MGH, an Mg^2+^ probe modified with a HaloTag ligand. (b) Absorption spectra of 10 μM MGH in the presence or absence of 100 mM Mg^2+^ (100 mM HEPES buffer, 115 mM KCl, 20 mM NaCl, pH 7.4, 37 °C). (c) The emission spectra of 1 μM MGH in the presence of Mg^2+^ (100 mM HEPES, 115 mM KCl, 20 mM NaCl, pH 7.4, 37 °C). [Mg^2+^] = 0, 0.01, 0.02, 0.04, 0.1, 0.2, 0.4, 1, 2, 4, 10, 20, 40, and 100 mM. *λ*_ex_ = 515 nm. (d) Mg^2+^-titration curve of MGH emission at 538 nm (*λ*_ex_ = 515 nm). The error bars show the SD (*n* = 3).

In order to visualize local Mg^2+^ dynamics in living cells for a long time, we designed and synthesized a new Mg^2+^ sensor, MGH, which covalently labels HaloTag (Fig. 1a). HaloTag is a modified haloalkane dehalogenase derived from *Rhodococcus*, and it quickly forms a covalent bond with specific ligands, including a chloroalkyl group.^31^ HaloTag can be expressed as a fusion protein with a localization signal peptide in mammalian cells. Thus, MGH, which involves a HaloTag ligand, was expected to be localized in specific target organelles, since similar strategies have previously been exploited to localize various small-molecule metal ion sensors in specific organelles.^22^–^24^

Here, magnesium green was adopted for Mg^2+^ sensing due to its suitable dissociation constant for Mg^2+^ (*K*_d_ = 1 mM), making it able to detect [Mg^2+^]_i_ changes.^32^ The probe was designed so that the fluorescence intensity of its Mg^2+^-unbound state is suppressed by photo-induced electron transfer (PeT),^33^–^35^ and it emits strong fluorescence *via* binding to Mg^2+^.

MGH is composed of three building blocks for synthesis: the Mg^2+^ chelator part, the fluorophore part, and the HaloTag ligand part. These components were individually synthesized, and were then conjugated to facilitate the synthetic process and optimization of each part. The Mg^2+^ chelator part (compound 4) was synthesized from *o*-aminophenol *via* S_N_2 reaction with chloroacetic acid, Fischer esterification, nitration, and alkaline hydrolysis (Scheme S1†).^36^ To improve the cell membrane permeability, compound 4 was then derivatized to tris-acetoxymethyl (AM) ester, which is quickly deprotected by endogenous intracellular esterases.^37^ In the fluorophore part, an alkynyl moiety of compound 10 was introduced at the 4-position of the xanthene ring of 5-carboxyfluorescein *via* the Mannich reaction (Scheme S2†).^38^ Then, the secondary amine, as well as the phenolic hydroxy groups, was modified with an acetyl group to avoid PeT from the free amino group to the fluorophore. Compound 15 was afforded *via* the amide condensation reaction of the chelator moiety (compound 6) and chromophore (compound 11), and then it was conjugated with the HaloTag ligand (compound 14) (Scheme S3†) *via* Cu(i)-catalyzed azide–alkyne cycloaddition to produce MGH(AM) (Scheme S4†). MGH(AM) was used for live cell imaging due to its sufficient cell membrane permeability. For the various *in vitro* spectral measurements, the deprotected probe MGH was synthesized *via* the alkaline hydrolysis of MGH(AM).

### Spectroscopic properties of MGH and HaloTag-MGH

The absorption and emission spectra of MGH were measured in 100 mM HEPES buffer (pH 7.4) containing 115 mM KCl and 20 mM NaCl. Although the absorption spectra of MGH did not change significantly with 100 mM Mg^2+^ (Fig. 1b), the fluorescence intensity of MGH considerably increased as the Mg^2+^ concentration was increased (*Φ*_free_ = 0.19, *Φ*_bound_ = 0.56, Fig. 1c). From the fluorescence intensity change, the dissociation constant of MGH for Mg^2+^ was calculated to be 1.3 mM (Fig. 1d), which is a suitable value for imaging [Mg^2+^]_i_ changes. The metal ion selectivity of MGH, shown in Fig. S1c,† was similar to that of other *o*-amino-phenol-*N*,*N*,*O*-triacetic acid (APTRA)-based fluorescent probes.^39^ For live-cell Mg^2+^ imaging, the ability of the probe to discriminate changes in intracellular Ca^2+^ concentration ([Ca^2+^]_i_) is quite important. MGH bound to Ca^2+^ more strongly (*K*_d_ (Ca^2+^) = 12 μM) than to Mg^2+^ (Fig. S1a and b†), although this had already been predicted at the stage of the probe design. Since [Ca^2+^]_i_ is roughly 100 nM and rises 10- to 100-fold during various cellular events,^40^ simultaneous monitoring of [Ca^2+^]_i_ with a Ca^2+^-specific probe is necessary to correctly evaluate intracellular Mg^2+^ dynamics with MGH. In addition, the pH sensitivity of live-cell imaging probes is another important factor for practical use. Concerning the pH effect, MGH scarcely showed a fluorescence intensity change in a physiological pH range (pH 7–8) (Fig. S1d†). These spectroscopic properties of MGH mostly corresponded with those of magnesium green, indicating that the attachment of the HaloTag ligand scarcely affected the metal ion-detecting properties of the Mg^2+^ sensor (Table 1 and Fig. S2†).

**Table 1:** Spectroscopic and coordination properties of MGH, HaloTag-MGH and magnesium green^*a*^

	*λ*_abs_ [nm]	*λ*_em_ [nm]	*ε* [cm^–1^ M^–1^]	*Φ*_free_ ^*b*^ (*Φ*_bound_)	*K*_d_ (Mg^2+^) [mM]	*K*_d_ (Ca^2+^) [μM]
MGH	515	538	77 000	0.19 (0.56)	1.3	12
HaloTag-MGH	517	540	78 000	0.21 (0.61)	0.67	7.5
Magnesium green	509	534	77 000	0.20 (0.56)	0.88	12

^*a*^Measured at 37 °C in 100 mM HEPES buffer, 115 mM KCl, 20 mM NaCl, pH 7.4.

^*b*^Relative fluorescence quantum yield determined using fluorescein (*Φ* = 0.85 in 0.1 M NaOH aq.) as a standard. *Φ*_free_ and *Φ*_bound_ denote the relative fluorescence quantum yield in the absence and presence of 100 mM Mg^2+^, respectively.

It is likely that spectroscopic properties of fluorescent probes are affected by environmental changes such as binding on the protein surface. Hence, we measured the spectroscopic properties of MGH after incubation with a purified HaloTag protein for 1 h at 37 °C. SDS-PAGE analysis showed almost all MGH covalently bound to HaloTag (Fig. S3†). Overall, the fluorescence properties of HaloTag-MGH were similar to those of MGH and magnesium green (Table 1 and Fig. S4†). We infer that, in response to Mg^2+^, HaloTag-MGH has almost the same dynamic range of as that of free MGH, which was derived from the hydrophilic spacer between the Mg^2+^ sensor part and the HaloTag ligand part. The hydrophilic spacer prevented the interaction between the chromophore and the protein surface of the HaloTag.^24^ Although the *K*_d_ values of HaloTag-MGH for Mg^2+^ and Ca^2+^ were enhanced approximately 2-fold, the affinity of HaloTag-MGH for Mg^2+^ was still appropriate for visualizing intracellular Mg^2+^ dynamics.

### Subcellular targeting of MGH and long-term imaging of Mg^2+^ in living cells

**Fig. 2:**
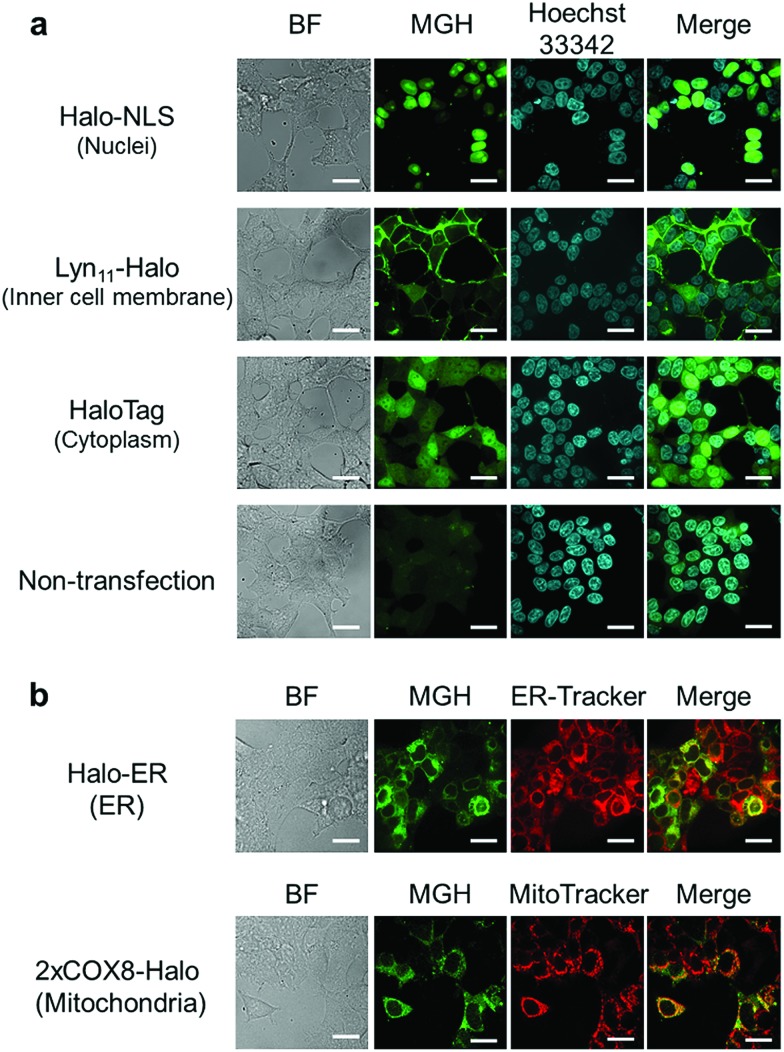
Subcellular localization of MGH in living cells. (a) Confocal fluorescence microscopic images of MGH localization in HEK293T cells transfected with a plasmid encoding Halo-NLS (nuclei), Lyn_11_-Halo (inner leaflet of cell membrane) or HaloTag (cytoplasm). Labeling reactions of fusion protein of Halo-NLS, Lyn_11_-Halo or HaloTag in HEK293T cells with 3 μM MGH(AM) and staining with 200 ng mL^–1^ Hoechst 33342 were performed for 1 h at 37 °C. (b) Confocal fluorescence microscopic images of MGH localization in ER or mitochondria matrix, which are surrounded by a lipid bilayer membrane. HEK293T cells transiently expressing Halo-ER or 2xCOX8-Halo were incubated with 3 μM MGH(AM) and either 200 nM ER-Tracker Red or 200 nM MitoTracker DeepRed for 1 h at 37 °C. Scale bar: 20 μm.

Here, we attempted to confirm the subcellular targeting of MGH to a variety of organelles. HEK293T cells were transfected with a plasmid encoding Halo-NLS, Lyn_11_-Halo, or HaloTag. NLS is a nuclear localization signal peptide^41^ and Lyn_11_ is a Lyn N-terminal sequence (GCIKSKGKDSA), which is used to target proteins to the inner leaflet of cell membrane.^42^,^43^ The transfected and non-transfected cells were incubated with 3 μM MGH(AM) for 1 h at 37 °C and were observed using a confocal spinning disk fluorescence microscope. In the case of the non-transfected cells, weak fluorescence was observed from the entire cell (Fig. 2a). However, in the transfected cells, strong fluorescence was observed from the target domains, such as nuclei, the cell membrane inner leaflet, or cytoplasm, without non-specific fluorescence signals (Fig. 2a).

Subsequently, MGH(AM) was added to HEK293T cells expressing HaloTag in the endoplasmic reticulum (ER) or the mitochondrial matrix. Since these organelles are covered by a lipid bilayer membrane, it was unclear whether fluorescent probes modified with AM esters could penetrate the second lipid bilayer membrane prior to the enzymatic hydrolysis of the AM esters by intracellular esterases. To express HaloTag in ER, the ER signal peptide and retention signal sequences (SEKDEL) were fused with HaloTag (Halo-ER). For mitochondrial matrix targeting, HaloTag was fused with the first 36 amino acids of subunit VIII of cytochrome c oxidase (COX8) in tandem to enhance the specificity of mitochondrial localization (2xCOX8-Halo).^44^ When the cells expressing Halo-ER or 2xCOX8-Halo were incubated with MGH(AM) for 1 h, the fluorescence of MGH was observed from the ER and mitochondria, respectively. The intended targeting of MGH to the ER and mitochondria was confirmed by the colocalization with the fluorescent signals of ER-Tracker Red and MitoTracker DeepRed, respectively (Fig. 2b). The results indicate that MGH(AM) diffused very quickly in living cells and accumulated inside of organelles surrounded by lipid bilayers, such as ER and the mitochondrial matrix, before enzymatic hydrolysis of the AM esters. However, it is likely that MGH mainly detects Ca^2+^ in the ER because the ER contains high concentrations of Ca^2+^ (hundreds of μM). Therefore, Mg^2+^-selective probes are necessary for the correct evaluation of changes in Mg^2+^ concentration in the ER.

**Fig. 3:**
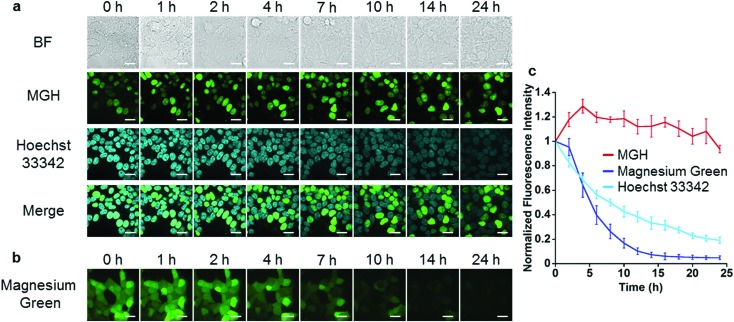
Comparison between MGH and magnesium green for long-term imaging. (a) Long-term Mg^2+^ imaging with MGH localized in nuclei for 24 h. HEK293T cells transiently expressing Halo-NLS were incubated with 3 μM MGH(AM) and 200 ng mL^–1^ Hoechst 33342 for 1 h at 37 °C. (b) Long-term Mg^2+^ imaging with magnesium green for 24 h. HEK293T cells were incubated with 1 μM magnesium green(AM) for 45 min at 37 °C. Scale bar: 20 μm. (c) Time-dependent changes of the relative fluorescence intensity of MGH, magnesium green or Hoechst 33342 in a single cell. The error bars denote SD (*n* = 4).

Next, long-term Mg^2+^ imaging in living cells was attempted by using the nucleus-localized MGH. Commercially available small-molecule Mg^2+^ probes, such as magnesium green, are not suitable for long-term imaging owing to the extracellular leakage *via* anion transporters.^12^,^13^ We also confirmed that the fluorescence of magnesium green gradually decreased in a time-dependent manner, and completely disappeared after 24 h (Fig. 3b and c). In contrast, the fluorescence of nucleus-localized MGH was continuously detected for 24 h (Fig. 3a and c). The fluorescence signals of Hoechst 33342, which is a membrane-permeable cationic dye, gradually decreased, probably due to diffusion of the dye (Fig. 3a and c). These results indicated that MGH enables imaging of Mg^2+^ dynamics for more than 1 day.

### Detection of Mg^2+^ extrusion through CNNM4

**Fig. 4:**
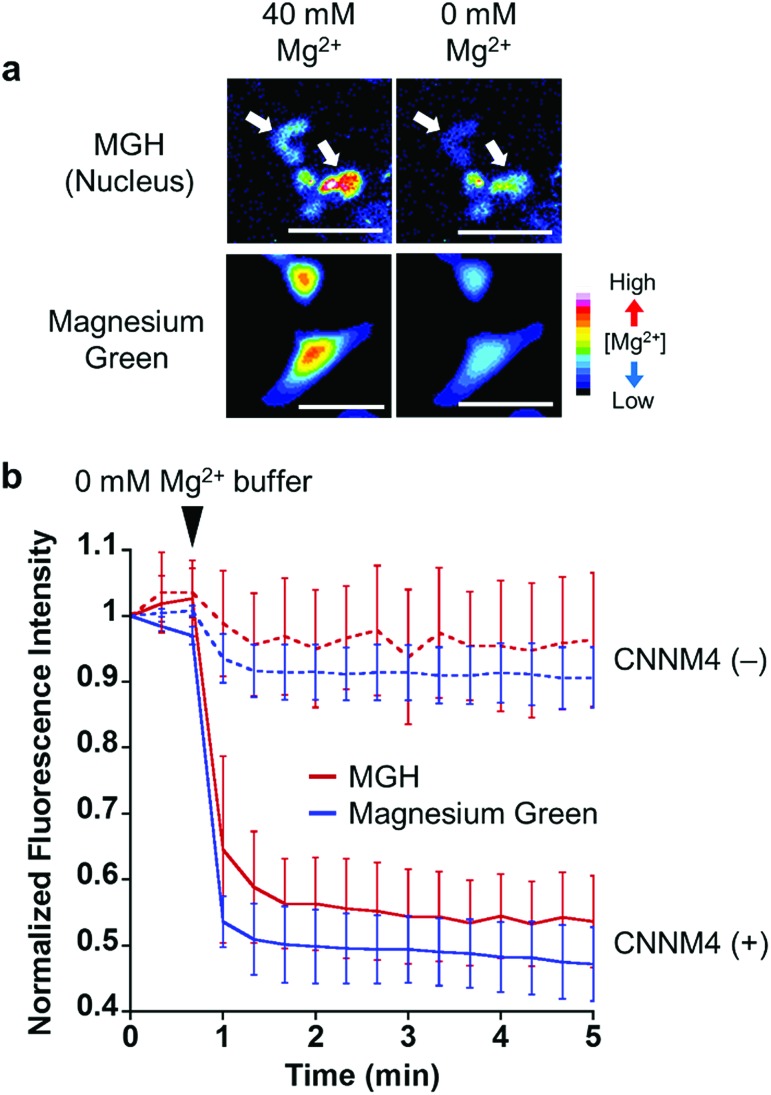
Visualization of Mg^2+^ extrusion *via* Mg^2+^ transporter, CNNM4, with MGH. (a) Epifluorescence microscopic images of Mg^2+^ extrusion with MGH or magnesium green. HEK293 cells transfected with CNNM4-FLAG and Halo-NLS were incubated with Mg^2+^-loading buffer (78.1 mM NaCl, 5.4 mM KCl, 1.8 mM CaCl_2_, 40 mM MgCl_2_, 5.5 mM glucose, 5.5 mM HEPES-KOH, pH 7.4), including 5 μM MGH(AM) or 2 μM magnesium green(AM), for 1 h at 37 °C. These cells were subjected to Mg^2+^ depletion 1 min after the imaging started. Scale bar: 40 μm. (b) The normalized fluorescence intensity of MGH or magnesium green in HEK293 cells subjected to Mg^2+^ depletion. The error bars denote SD (MGH: *n* = 6, magnesium green: *n* = 10).

To examine the response of nucleus-localized MGH to Mg^2+^, we performed a Mg^2+^ extrusion experiment using a Mg^2+^ transporter, ancient conserved domain protein/cyclin M4 (CNNM4). CNNM4 is strongly expressed in the intestinal epithelium and can extrude Mg^2+^ by stimulating Na^+^/Mg^2+^ exchange.^45^ CNNM4-FLAG and Halo-NLS were co-expressed in HEK293 cells, in which the transient expression of CNNM4 was confirmed by western blot analysis (Fig. S5b†), and the cells were incubated with MGH(AM) in 40 mM Mg^2+^ buffer. After 1 h, the extracellular solution was exchanged with a Mg^2+^-free buffer to artificially promote Mg^2+^ extrusion *via* CNNM4, and time-lapse fluorescence microscopic images were captured. The fluorescence intensity of nucleus-localized MGH, as well as magnesium green, immediately decreased after Mg^2+^ depletion from the extracellular buffer (Fig. 4 and S5a†). The fluorescence of MGH and magnesium green in the control cells not overexpressing CNNM4 remained constant after the addition of the Mg^2+^-free buffer. Thus, it was confirmed that MGH maintained a quick fluorescence response to [Mg^2+^]_i_ change. Since MGH showed promise for long-term imaging, the durability of the response to Mg^2+^ was investigated. The Mg^2+^ extrusion experiment was conducted after 24 h of MGH(AM) loading to the transfected HEK293 cells. As a result, the fluorescence of MGH quickly decreased after exchanging a buffer containing 40 mM Mg^2+^ with the Mg^2+^-free buffer (Fig. S5c and d†). This result showed that MGH retained the Mg^2+^ responsivity for 24 h in living cells.

### Long-term monitoring of intracellular Mg^2+^ dynamics during apoptosis

Taking advantage of the applicability to long-term imaging, MGH was applied to investigate intracellular Mg^2+^ dynamics during apoptosis induced by the anti-Fas antibody and cycloheximide (CHX). So far, anti-Fas antibody-mediated [Mg^2+^]_i_ increase in apoptotic B cells was fragmentally detected for 16 h with a ratiometric Mg^2+^ probe, Mag-indo-1, by flow cytometric analysis.^11^ However, this method did not provide detailed information regarding the timing of Mg^2+^ mobilization along with the apoptotic process. On the other hand, use of MGH simultaneously enables real-time monitoring of [Mg^2+^]_i_ and changes in cellular morphology over a long time period. However, MGH exhibits an increase in fluorescence intensity without a shift in wavelength. This means that the fluorescence intensity depends not only on Mg^2+^ concentration but also on other factors such as probe concentration and light intensity. Particularly, probe concentration must be changed during apoptosis, because apoptotic cells show shrinking. Therefore, to exclude the influence of such factors, an internal standard was adopted for calibration. As the internal standard, a red fluorescent HaloTag ligand, Halo-TMR, was used to label HaloTag, and the fluorescence intensity ratio of MGH and Halo-TMR was calculated.

**Fig. 5:**
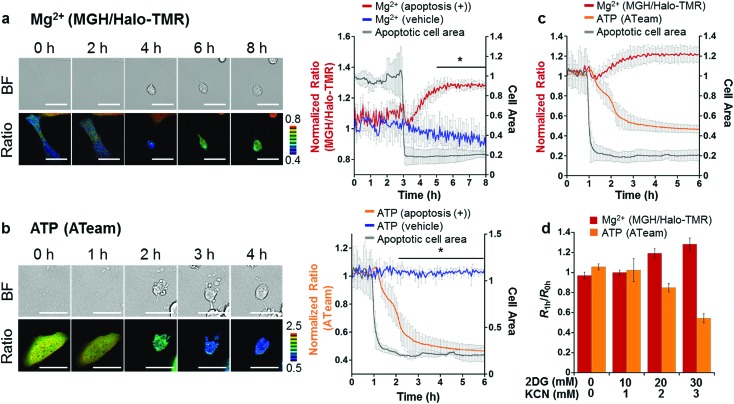
Confocal fluorescence imaging of (a) Mg^2+^ and (b) ATP dynamics with progression of apoptosis in HeLa cells. (a) HeLa cells were transfected with a plasmid encoding HaloTag. Fluorescence of 3 μM MGH(AM) was normalized with 50 nM Halo-TMR to exclude the influence of changes in probe concentration during apoptosis. (b) HeLa cells were transfected with a plasmid encoding ATeam, a FRET-based ATP sensor. Apoptosis inducers were anti-Fas antibody (250 ng mL^–1^) and cycloheximide (10 μg mL^–1^). Scale bar: 40 μm. Statistical analyses were performed using the Student’s *t*-test. *P < 0.05. The error bars denote SD (*n* = 3). (c) Correlation between [Mg^2+^] and [ATP] in apoptosis-induced HeLa cells. Each ratio change was individually recorded and plotted from 1 h before cell shrinkage. (Mg^2+^: *n* = 3, ATP: *n* = 3). (d) Dose-dependent effect of 2DG and KCN on [Mg^2+^]_i_ and ATP concentration. Ratio changes for Mg^2+^ and ATP were individually recorded. The error bars denote SD (*n* = 5).

HeLa cells transiently expressing HaloTag in the cytoplasm were labeled with MGH(AM) for 30 min and subsequently Halo-TMR for 15 min. Then, the time-lapse imaging experiment was started, and anti-Fas antibody and CHX were added after 30 min. Alexa Fluor™ 350 annexin V conjugate, which binds phosphatidylserine-binding protein, was also added to identify apoptotic cells. As a result, the fluorescence signal ratio of MGH and Halo-TMR increased after the apoptotic cell shrinkage, and then plateaued (Fig. 5a). Quantitative analysis showed that [Mg^2+^]_i_ increased from approximately 1 to 1.8 mM during apoptosis (Fig. S6†).

We also checked the validity of our ratiometric imaging method with Halo-TMR as the internal standard. During long-term microscopic imaging experiments, fluorescent probes are likely to cause photobleaching. Since the photostability of fluorescent probes is dependent on the dye skeletons, we measured the photostability of MGH and Halo-TMR using a fluorometer under continuous irradiation at 490 and 550 nm, respectively (Fig. S7†). The fluorescence intensity of MGH decreased slightly during long-term irradiation as compared with that of Halo-TMR. Indeed, this result indicated that the fluorescence ratio of MGH/Halo-TMR may change without changes in [Mg^2+^]_i_ during long-term imaging experiments. Actually, long-term microscopic imaging data demonstrated that the fluorescence ratio of MGH/Halo-TMR in HaloTag-expressing HeLa cells without the addition of apoptosis inducers continued to decrease slightly for several hours (Fig. 5a), and this small ratio change could be interpreted as the photobleaching of MGH. However, in apoptotic cells, the ratio tended to increase. Thus, we concluded that the fluorescence ratio change in the apoptotic cells properly reflected the changes in [Mg^2+^]_i_. Moreover, in another control experiment, the photostability of Halo-TMR during apoptosis was examined by calculating the ratio of Halo-OG to Halo-TMR (Fig. S8†). The fluorescence ratio of the two dyes remained constant during apoptosis. All of these results supported the adequacy of our ratiometric system.

As shown in Table 1, APTRA-based Mg^2+^ probes respond to high concentrations of Ca^2+^. It is suggested that Ca^2+^ release from ER through the IP_3_ receptor to the cytosol is one of the key apoptotic processes.^46^ Therefore, discrimination between the concentration changes of Ca^2+^ and Mg^2+^ is necessary. The change in [Ca^2+^]_i_ during apoptosis was visualized with a genetically encoded Ca^2+^-specific fluorescent probe, R-GECO, which shows a Ca^2+^-dependent increase in red fluorescence (*K*_d_ (Ca^2+^) = 480 nM).^47^ R-GECO barely showed a fluorescence response to the Mg^2+^ introduced by the ionophore 4-bromo-A23187, in contrast to the increase in the fluorescence intensity of MGH, demonstrating sufficient specificity for Ca^2+^ (Fig. S9†). R-GECO and HaloTag were transiently expressed in HeLa cells, and the transfected cells were stained with green fluorescent Halo-OG to accurately evaluate Ca^2+^ dynamics by monitoring the fluorescence ratio of R-GECO/Halo-OG. A sharp spike was observed immediately after the addition of apoptosis inducers, which was followed by a slight increase during apoptosis (Fig. S10b†). This large spike was not observed by monitoring the MGH/Halo-TMR ratio, indicating that MGH has a sufficiently low affinity for Ca^2+^ in the physiological range of cytosolic [Ca^2+^]_i_ during apoptosis (Fig. S10a†). Thus, the fluorescence response of MGH after the apoptotic cell shrinkage was ascribed to the increase of [Mg^2+^]_i_, not [Ca^2+^]_i_.

Another concern related to the increase in the fluorescence ratio of MGH/Halo-TMR is equilibration of intracellular and extracellular metal ions due to the loss of cell membrane integrity in the late stages of apoptosis.^58^ During apoptosis imaging, we observed further significant increases in the fluorescence ratios of both MGH/Halo-TMR and R-GECO/Halo-OG following increases in the ratios after apoptotic cell shrinkage (Fig. S10,† blue squares). This large fluorescence response during the late apoptotic stage is thought to reflect the loss of cell membrane integrity and the influx of extracellular metal ions, particularly Ca^2+^ (Ca^2+^ concentration in DMEM is approximately 1.8 mM).

To elucidate the mechanism of [Mg^2+^]_i_ increase during apoptosis, we focused on the correlation between [Mg^2+^]_i_ and intracellular ATP concentration; since most ATP forms a complex with Mg^2+^ in cells,^1^ [Mg^2+^]_i_ is likely to be affected by ATP concentration. In fact, it has been reported that various stimulations, such as cyanide,^48^ fructose,^49^ and anoxia,^50^ trigger an increase in [Mg^2+^]_i_ with a decrease in intracellular ATP or degradation of ATP to ADP or AMP. These reports suggested a close relationship between Mg^2+^ and ATP levels. Another previous study showed that intracellular ATP concentration considerably decreased at the late stage of apoptosis.^51^ Hence, the time course of ATP concentration change in apoptotic HeLa cells was visualized using a caspase-resistant version of ATeam,^52^ a genetically encoded fluorescence resonance energy transfer (FRET)-based ATP sensor.^53^ As a result, the ATP level significantly decreased in apoptotic cells after cell shrinkage (Fig. 5b).

Although the simultaneous imaging of [Mg^2+^]_i_ and ATP concentration in apoptotic cells was desirable, it was not possible because the excitation and emission wavelengths of MGH and ATeam largely overlap. Therefore, the fluorescence ratio changes for [Mg^2+^]_i_ and intracellular ATP concentration were plotted from 1 h before cell shrinkage (Fig. 5c). The time-course graphs of both concentrations indicated that [Mg^2+^]_i_ and intracellular ATP concentration were almost inversely correlated.

Was this [Mg^2+^]_i_ increase induced by the decrease in intracellular ATP concentration in apoptotic cells? We hypothesized that the increase in Mg^2+^ levels arose from disassociation of Mg^2+^ from ATP. To confirm this hypothesis, intracellular ATP concentration was lowered by adding 2-deoxyglucose (2DG) and potassium cyanide (KCN), which are inhibitors of glycolysis and oxidative phosphorylation, respectively.^53^ Incubation with the inhibitors for 1 h induced a decrease in ATP concentration depending on the inhibitor concentration, with a concomitant increase of [Mg^2+^]_i_ (Fig. 5d). This relationship between Mg^2+^ and ATP concentrations suggested that the [Mg^2+^]_i_ increase after apoptotic cell shrinkage was caused by the dissociation of Mg^2+^ from ATP. Our results indicated that Mg-ATP was a possible source of free Mg^2+^ in apoptotic cells. Importantly, a variety of endonucleases involved in DNA fragmentation during apoptosis requires Mg^2+^ to digest DNA.^54^ The increase in Mg^2+^ levels after the cell shrinkage may be relevant to endonuclease activities. Further studies will be necessary to investigate the role of Mg^2+^ following apoptotic cell shrinkage.

## Discussion

Fluorescence imaging with higher temporal and spatial resolution is a crucial technique for the analysis of target molecules. To elucidate the intracellular dynamics and physiological roles of free Mg^2+^, this imaging system requires the long-term retention and localization of fluorescent Mg^2+^ probes in living cells. However, existing Mg^2+^ probes have not been suitable for such experiments, especially for long-term imaging. To overcome this problem, we developed a combined system of a novel small-molecule Mg^2+^ probe, MGH, and a HaloTag, which is genetically localized in a variety of organelles. This noteworthy feature improved the spatiotemporal resolution for detecting intracellular Mg^2+^ dynamics, enabling the investigation of unexplored phenomena related to Mg^2+^. Recently, Buccella and co-workers reported a similar imaging system combining a ratiometric Mg^2+^ probe, Mag-S-Tz, and HaloTag.^25^ This probe was also localized at the target subcellular compartments in living cells after the labeling of a strained bicyclononyne ligand to HaloTag and the subsequent fluorogenic click reaction between Mag-S-Tz and the bicyclononyne. However, this two-step strategy requires more than 4 h to start the imaging experiment, and long-term imaging has not been achieved. Therefore, MGH is the first imaging probe to have demonstrated long-term time-lapse imaging of local Mg^2+^ dynamics in living cells for 24 h.

Apoptosis is a process that takes several hours. Therefore, long-term imaging is essential for the clarification of intracellular free Mg^2+^ dynamics in apoptotic cells. In the 1990s, some groups showed a [Mg^2+^]_i_ increase during apoptosis.^10^,^11^ However, more detail on the mechanisms and timing of the [Mg^2+^]_i_ increase have been unclear owing to the lack of long-term Mg^2+^ imaging methods. MGH had suitable properties for long-term monitoring of [Mg^2+^]_i_ change during apoptosis. However, MGH showed a simple fluorescence in response to the change of [Mg^2+^] without an excitation or emission wavelength shift. This fluorescence spectral property was not ideal for the analysis of apoptotic cells because the fluorescence intensity is considerably affected by the probe concentration change during apoptosis. This problem was solved by introducing Halo-TMR as an internal standard, and thus calculating the signal ratio between MGH and Halo-TMR. Ratiometric two-fluorophore sensing systems have been employed in Ca^2+^ and Zn^2+^ imaging.^5^,^55^ In addition, extracellular leakage of the internal standard during long-term imaging was prevented by using Halo-TMR in HaloTag-expressing cells.

The data analysis of Mg^2+^ and ATP imaging with MGH and ATeam suggested that the [Mg^2+^]_i_ increase after apoptotic cell shrinkage was caused by the dissociation of Mg^2+^ from ATP. The inverse correlation of [Mg^2+^]_i_ and ATP concentration in the analysis of the dose-dependent effect of 2DG and KCN strongly supports the concept that Mg-ATP is the main resource for the Mg^2+^ increase after cell shrinkage during apoptosis. Although a close correlation between [Mg^2+^]_i_ and ATP levels has been previously reported, further evidence was suggested by biochemical characterization after cell lysis without direct visualization of both molecular behaviors in cells.^49^ Therefore, our result provides strong evidence regarding the [Mg^2+^]_i_ increase as a consequence of intracellular ATP depletion.

In HeLa cells, the intracellular ATP concentration was estimated at 3–4 mM,^56^,^57^ and it was reported that the intracellular ATP concentration in human T cells was almost fully depleted during apoptosis.^51^ If all of the discharged ATP released Mg^2+^ in cells, [Mg^2+^]_i_, which is maintained at the hundred-micromolar level in living cells, would increase to the several-millimolar level. However, [Mg^2+^]_i_ increased by only approximately 0.8 mM during apoptosis (Fig. S6†). This result suggested that increased Mg^2+^ was buffered by Mg^2+^ transporters or channels. Recently, some Mg^2+^ transporters and channels were identified in the cell membrane and in the membrane of intracellular organelles (*e.g.* mitochondria and Golgi).^1^ These Mg^2+^ transport mechanisms may contribute to the regulation of intracellular Mg^2+^ homeostasis. Since MGH can be localized on a Mg^2+^ transporter by tandemly expressed HaloTag, Mg^2+^ influx *via* Mg^2+^ transporters would be visualized using such organelle-localized probes. Further studies using MGH should lead to the elucidation of Mg^2+^-buffering mechanisms in apoptotic cells.

## Conclusions

In conclusion, we developed a novel Mg^2+^ probe, MGH, which covalently binds to HaloTag protein expressed in various cellular compartments. The conjugation of MGH to HaloTag dramatically suppressed the extracellular leakage of MGH, and the Mg^2+^ sensing ability of MGH was retained for 24 h. These noteworthy properties were successfully applied to long-term visualization of intracellular Mg^2+^ dynamics during apoptosis. The results demonstrated an increase in Mg^2+^ concentration after apoptotic cell shrinkage. Subsequent experiments utilizing a FRET-based ATP sensor showed that dissociation of Mg^2+^ from ATP in apoptotic cells was the source of the increase of Mg^2+^ concentration. Thus, long-term imaging with a HaloTag-coupled Mg^2+^ probe provided precise information regarding intracellular Mg^2+^ dynamics during apoptosis. This study includes the molecular design of a cell-functional probe that involves a synthetic organic molecule and a genetically encoded protein. In addition, the findings in this study should contribute to the understanding of Mg^2+^-related biology, which includes very wide areas in biology and medicine, and thus the results strongly suggest that this new methodology will be a robust tool for studying Mg^2+^ dynamics in living systems and diseases.

## Experimental section

### Live-cell fluorescence imaging of MGH localization

HEK293T cells maintained in 10% FBS in DMEM at 37 °C in 5% CO_2_ were transfected with pcDNA-3.1-(+)-Halo-NLS, pcDNA-3.1-(+)-Lyn_11_-Halo, pcDNA-3.1-(+)-HaloTag, pKmc-2xCOX8-Halo or pmKate2-Halo-ER plasmids using Lipofectamine 3000, and the cells were incubated at 37 °C for 24 h. Then, the cells were washed three times with HBSS and incubated in FBS-free DMEM containing 3 μM MGH(AM) for 1 h in a CO_2_ incubator. After washing with HBSS, fluorescence images were captured in DMEM containing 10% FBS using a confocal fluorescence microscope at 37 °C.

### Mg^2+^ extrusion experiments

HEK293 cells were transfected with pCMV-CNNM4-FLAG and pcDNA-3.1-(+)-Halo-NLS using Lipofectamine 2000, and the cells were incubated at 37 °C for 24 h. Then, the cells were incubated with Mg^2+^-loading buffer (78.1 mM NaCl, 5.4 mM KCl, 1.8 mM CaCl_2_, 40 mM MgCl_2_, 5.5 mM glucose, 5.5 mM HEPES-KOH, pH 7.4), including 5 μM MGH(AM) or 2 μM magnesium green, for 1 h at 37 °C. The cells were rinsed once with loading buffer and viewed using an epifluorescence microscope (IX81 equipped with a DP30BW camera and a USH-1030L mercury lamp; Olympus). Fluorescence was measured every 20 s (excitation at 470–490 nm and emission at 505–545 nm) under the control of the MetaMorph software (Molecular Devices). Then, the buffer was changed to a Mg^2+^-free buffer (MgCl_2_ in the loading buffer was replaced with 60 mM NaCl). The Mg^2+^ extrusion experiment 24 h after loading MGH(AM) to HEK293 cells was performed as follows. HEK293 cells were firstly transfected with pcDNA-3.1-(+)-Halo-NLS, and the cells were incubated at 37 °C for 24 h. The cells were incubated with 5 μM MGH(AM) in DMEM (FBS free) for 1 h at 37 °C, and then the cells were transfected with pCMV-CNNM4-FLAG at 37 °C for 24 h. After incubation with Mg^2+^-loading buffer for 30 min, the cells were rinsed once with loading buffer and viewed using an epifluorescence microscope.

### Fluorescence imaging during apoptosis

#### a Mg^2+^

HeLa cells maintained in 10% FBS in DMEM at 37 °C in 5% CO_2_ were transfected with the pcDNA-3.1-(+)-HaloTag plasmid using Lipofectamine 3000. After 24 h, the cells were washed twice with HBSS, incubated with 3 μM MGH(AM) for 30 min, and then treated with 50 nM Halo-TMR for 15 min at 37 °C under 5% CO_2_. After washing with HBSS, fluorescence images were captured in DMEM containing 10% FBS and 5 μL AnnexinV (Alexa Fluor 350) using a confocal fluorescence microscope at 37 °C. Anti-Fas antibody (250 ng mL^–1^) and cycloheximide (10 μg mL^–1^) were added 30 min after the imaging started. Change in [Mg^2+^]_i_ was determined using the following equation:[Mg^2+^]_i_ = *K*_d_*Q*(*R* – *R*_min_)/(*R*_max_ – *R*),where *R* is the signal ratio of MGH/Halo-TMR, *R*_min_ is the minimum value of *R*, *R*_max_ is the maximum value of *R*, *Q* is the signal ratio of Halo-TMR under minimum Mg^2+^ concentration to Halo-TMR under maximum Mg^2+^ concentration, and *K*_d_ is 0.67 mM for Halo-MGH. *R*_min_ and *R*_max_ were calculated after imaging experiments as follows. The apoptotic cells were washed with Mg^2+^- and Ca^2+^-free HHBSS buffer, then permeabilized with 20 μg mL^–1^ digitonin in Mg^2+^- and Ca^2+^-free HHBSS for 5 min. After washing the cells with Mg^2+^- and Ca^2+^-free HHBSS buffer, *R*_min_ was recorded by incubating the cells in Mg^2+^- and Ca^2+^-free HHBSS buffer containing 10 mM EDTA at 37 °C for 10 min. Then, *R*_max_ was recorded by incubating the cells in Ca^2+^-free HHBSS buffer containing 50 mM MgCl_2_ at 37 °C for 10 min.

#### b Ca^2+^

HeLa cells maintained in 10% FBS in DMEM at 37 °C in 5% CO_2_ were transfected with the pcDNA-3.1-(+)-HaloTag and CMV-R-GECO1 plasmids using Lipofectamine 3000. After 24 h, the cells were washed twice with HBSS and incubated with 100 nM Halo-OG for 30 min at 37 °C under 5% CO_2_. After washing with HBSS, fluorescence images were captured in DMEM containing 10% FBS and 5 μL AnnexinV (Alexa Fluor 350) using a confocal fluorescence microscope at 37 °C.

#### c ATP

HeLa cells maintained in 10% FBS in DMEM at 37 °C in 5% CO_2_ were transfected with the pcDNA-3.1-(+)-ATeam plasmid using Lipofectamine 3000. After 24 h, the cells were washed two times with HBSS and then fluorescence images were captured in DMEM containing 10% FBS and 5 μL AnnexinV (Alexa Fluor 680) using a confocal fluorescence microscope at 37 °C.

### Dose-dependent effect of 2DG and KCN

HeLa cells were transfected with ATeam or HaloTag plasmids. The cells expressing HaloTag were washed twice with HBSS, incubated with 3 μM MGH(AM) for 30 min, and then treated with 50 nM Halo-TMR for 15 min at 37 °C. After washing with HBSS, fluorescence images were captured in DMEM at 37 °C for 1 h. The 2DG and KCN were added 10 min after the imaging started.

## Conflicts of interest

There are no conflicts to declare.

## Supplementary Material

Supplementary information
