# Supplementary material for: Visualization of long-term Mg2+ dynamics in apoptotic cells using a novel targetable fluorescent probe
Source: Chem Sci. 2017 Oct 20;8(12):8255–64. doi: 10.1039/c7sc03954a (PMC5858021; doi:10.1039/c7sc03954a)
Supplement: Supplementary file 1 [file SC-008-C7SC03954A-s001.pdf]

Electronic Supplementary Material (ESI) for Chemical Science.
This journal is © The Royal Society of Chemistry 2017

Supporting Information

Visualization of Long-term Mg^{2+} Dynamics in Apoptotic Cells with a Novel Targetable Fluorescent Probe

Authors: Yusuke Matsui,[a] Yosuke Funato,[b] Hiromi Imamura,[c] Hiroaki Miki,[b] Shin Mizukami,*[d] and Kazuya Kikuchi*[ae]

[a] Department of Material and Life Science, Graduate School of Engineering, Osaka University, Suita, Osaka 565-0871, Japan.
[b] Department of Cellular Regulation, Research Institute for Microbial Diseases, Osaka University, Suita, Osaka 565-0871, Japan.
[c] Graduate School of Biostudies, Kyoto University, Kyoto 606-8501, Japan.
[d] Institute of Multidisciplinary Research for Advanced Materials, Tohoku University, Sendai, Miyagi 980-8577, Japan.
[e] Immunology Frontier Research Center, Osaka University, Suita, Osaka 565-0871, Japan.
*Correspondence authors: Shin Mizukami: shin.mizukami@m.tohoku.ac.jp, Kazuya Kikuchi: kkikuchi@mls.eng.osaka-u.ac.jp

Table of Contents

1. Supplementary Figures 2
2. Supporting Methods 10
   Materials and instruments 10
   Fluorometric analysis 11
   Determination of dissociation constants 11
   Metal ion selectivity study 12
   Construction of plasmids 12
   Preparation of HaloTag protein 14
   Detection of protein labeling by SDS-PAGE 14
   Western blot analysis 14
   Cell culture 15
   Metal ion responsivity of MGH and R-GECO in HeLa cells 15
   Chemical synthesis 16
3. Supplementary References 30

1. Supplementary Figures

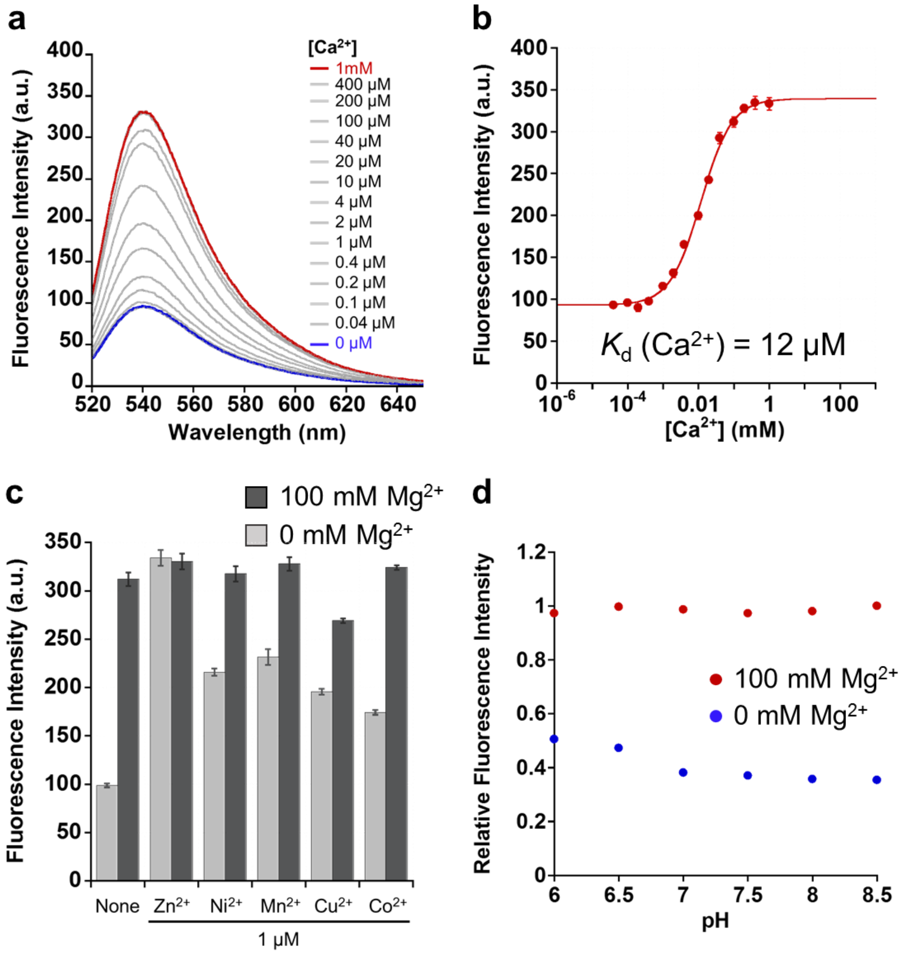

Fig. S1. (a) Emission spectra of 1 μM MGH in the presence of Ca^{2+} (100 mM HEPES, 115 mM KCl, 20 mM NaCl, pH 7.4, 37 °C). [Ca^{2+}] = 0, 0.04, 0.1, 0.2, 0.4, 1, 2, 4, 10, 20, 40, 100, 200, 400, 1000 μM. λ_{ex} = 515 nm. (b) Ca^{2+}-titration curve of MGH emission at 538 nm. (c) Metal ion selectivity for 1 μM MGH in the presence and absence of 100 mM Mg^{2+} (100 mM HEPES buffer, 115 mM KCl, 20 mM NaCl, pH 7.4, 37 °C). Zn^{2+}, Ni^{2+}, Mn^{2+}, Cu^{2+} or Co^{2+} were added to a final concentration of 1 μM. (d) Effect of the pH on the fluorescence intensity of MGH in the pH range of 6.0–6.5 (in 100 mM MES buffer, 115 mM KCl, 20 mM NaCl) and 7.0–8.5 (in 100 mM HEPES buffer, 115 mM KCl, 20 mM NaCl), with or without 100 mM Mg^{2+}. The error bars denote SD (n = 3).

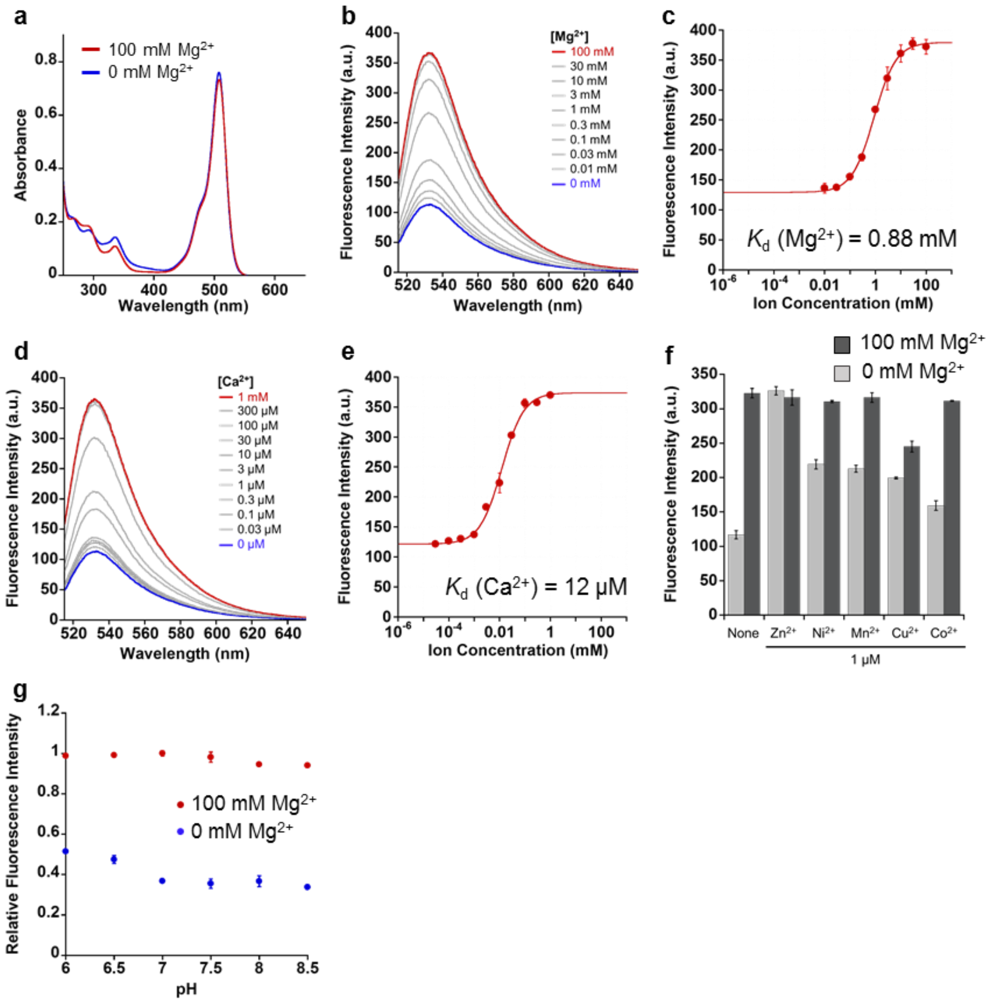

Fig. S2. (a) Absorption spectra of 10 μM Magnesium Green in the presence or absence of 100 mM Mg^{2+} (100 mM HEPES buffer, 115 mM KCl, 20 mM NaCl, pH 7.4, 37 °C). (b) Emission spectra of 1 μM Magnesium Green in the presence of Mg^{2+} (100 mM HEPES, 115 mM KCl, 20 mM NaCl, pH 7.4, 37 °C). [Mg^{2+}] = 0, 0.01, 0.03, 0.1, 0.3, 1, 3, 10, 30, 100 mM. λ_{ex} = 509 nm. (c) Mg^{2+}-titration curve of Magnesium Green emission at 534 nm (λ_{ex} = 509 nm). (d) Emission spectra of 1 μM Magnesium Green in the presence of Ca^{2+} (100 mM HEPES, 115 mM KCl, 20 mM NaCl, pH 7.4, 37 °C). [Ca^{2+}] = 0, 0.03, 0.1, 0.3, 1, 3, 10, 30, 100, 300, 1000 μM. λ_{ex} = 509 nm. (e) Ca^{2+}-titration curve of Magnesium Green emission at 534 nm (λ_{ex} = 509 nm). (f) Metal ion selectivity for 1 μM Magnesium Green in the presence and absence of 100 mM Mg^{2+} (100 mM HEPES buffer, 115 mM KCl, 20 mM NaCl, pH 7.4, 37 °C). Zn^{2+}, Ni^{2+}, Mn^{2+}, Cu^{2+} or Co^{2+} were added to a final concentration of 1 μM. (g) Effect of the pH on the fluorescence intensity of Magnesium Green between pH 6.0–6.5 (in 100 mM MES buffer, 115 mM KCl, 20 mM NaCl) and pH 7.0–8.5 (in 100 mM HEPES buffer, 115 mM KCl, 20 mM NaCl), with or without 100 mM Mg^{2+}. The error bars denote SD (n = 3).

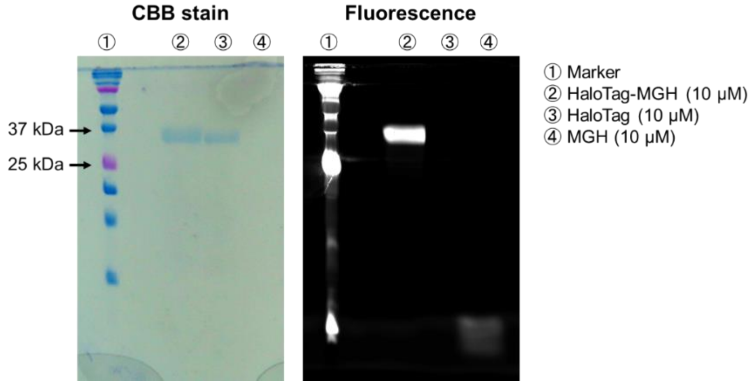

Fig. S3. SDS-PAGE analysis of complex of HaloTag and MGH. 40 μM HaloTag (33 kDa) and 30 μM MGH were incubated at 37 °C for 1 h. After incubation, the gel was analyzed by CBB stain (left) and fluorescence detection (right).

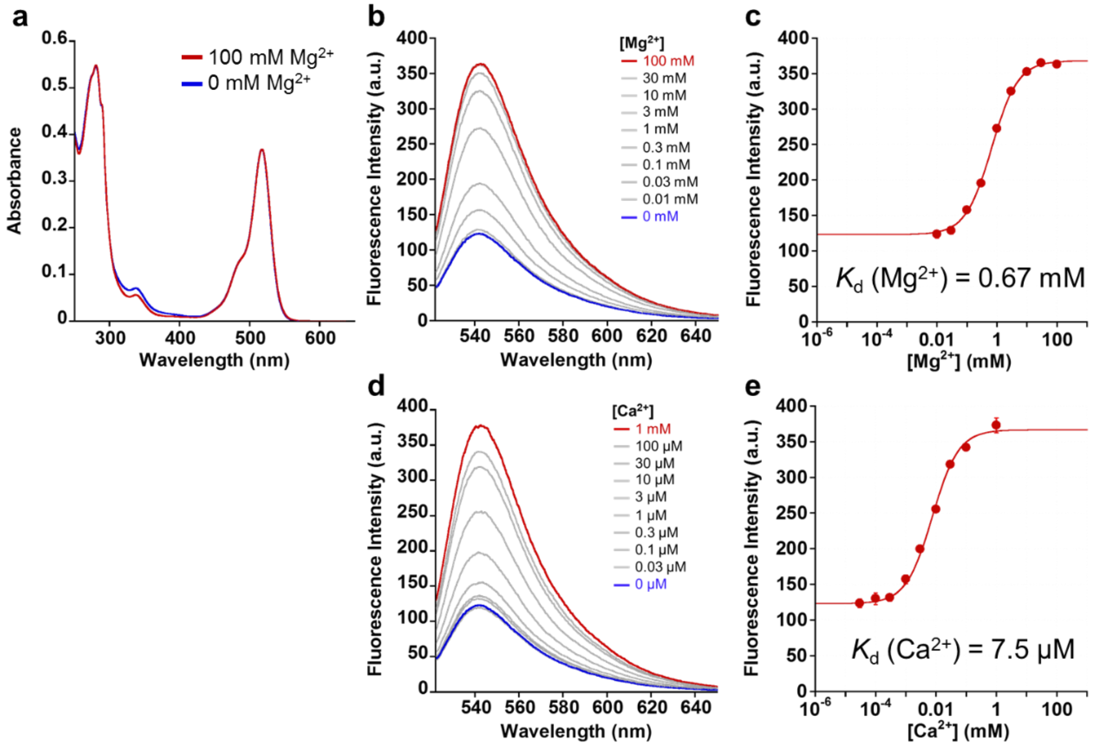

Fig. S4. (a) Absorption spectra of 5 μM HaloTag-MGH in the presence or absence of 100 mM Mg^{2+} (100 mM HEPES buffer, 115 mM KCl, 20 mM NaCl, pH 7.4, 37 °C). (b) Emission spectra of 1 μM HaloTag-MGH complex in the presence of Mg^{2+} (100 mM HEPES, 115 mM KCl, 20 mM NaCl, pH 7.4, 37 °C). [Mg^{2+}] = 0, 0.01, 0.03, 0.1, 0.3, 1, 3, 10, 30, 100 mM. λ_{ex} = 517 nm. (c) Mg^{2+}-titration curve of HaloTag-MGH emission at 540 nm (λ_{ex} = 517 nm). The error bars denote SD (n = 3). (d) Emission spectra of 1 μM HaloTag-MGH in the presence of Ca^{2+} (100 mM HEPES, 115 mM KCl, 20 mM NaCl, pH 7.4, 37 °C). [Ca^{2+}] = 0, 0.03, 0.1, 0.3, 1, 3, 10, 30, 100, 300, 1000 μM. λ_{ex} = 517 nm. (e) Ca^{2+}-titration curve of HaloTag-MGH emission at 540 nm (λ_{ex} = 517 nm). The error bars denote SD (n = 3).

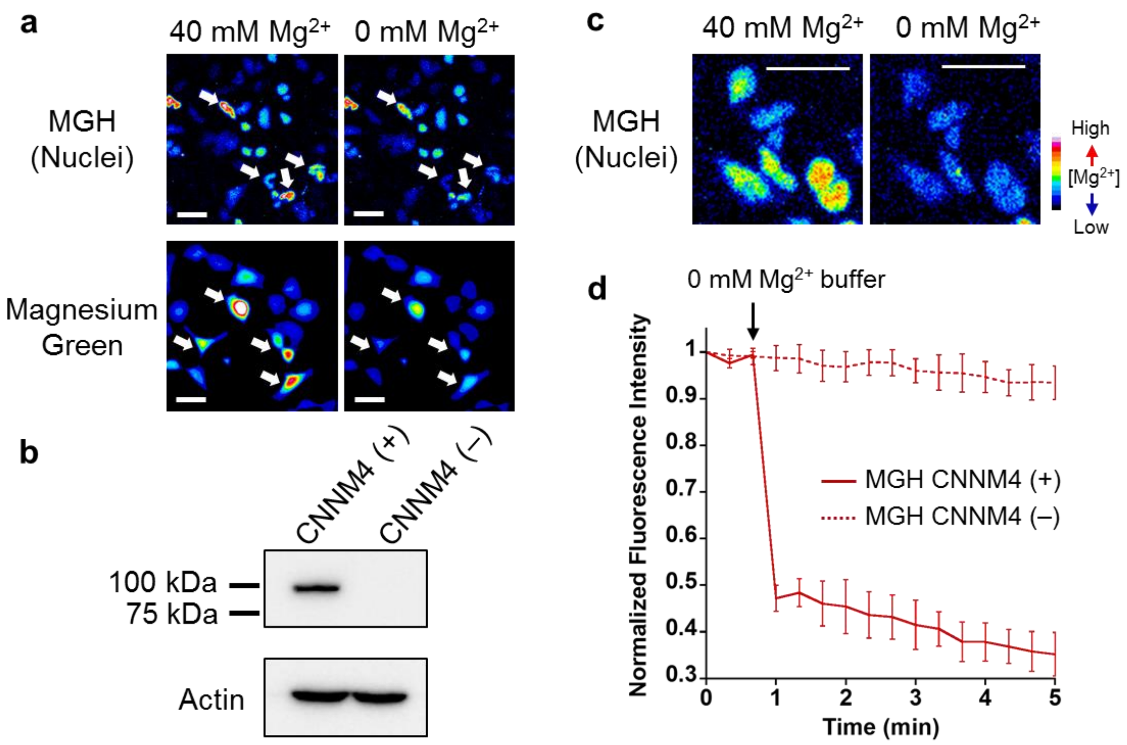

Fig. S5. (a) Large-field-of-view epifluorescence microscopic images in Mg^{2+} extrusion experiments shown in Fig. 4a. (b) Western blot analysis of CNNM4-FLAG (87 kDa) transiently expressed in HEK293 cells with anti-FLAG (top) or anti-actin (bottom) antibodies. (c) Epifluorescence microscopic images of Mg^{2+} extrusion after 24 h of MGH(AM) loading. HEK293 cells were firstly transfected with Halo-NLS. After 24 h, 5 µM MGH(AM) was loaded, then the cells were transfected with CNNM4-FLAG. These cells were subjected to Mg^{2+} depletion 1 min after imaging starting. Scale bar: 20 µm. (d) The normalized fluorescence intensity of MGH in HEK293 cells subjected to Mg^{2+} depletion after 24 h of the probe loading. The error bars denote SD (n = 5).

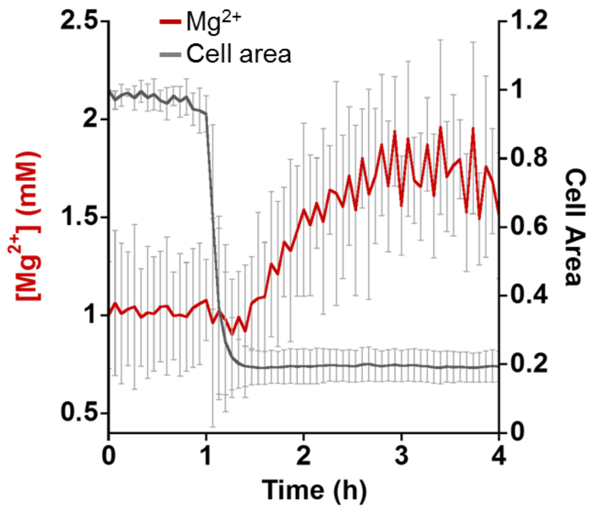

Fig. S6. Quantitative analysis of $[Mg^{2+}]_i$ changes during apoptosis. HaloTag-expressing HeLa cells labelled with MGH(AM) and Halo-TMR were subjected to apoptosis imaging under conditions similar to those in Fig. 5a. $[Mg^{2+}]_i$ during apoptosis was quantitatively analyzed by in situ calibration. Details are given in the Experimental section. The error bars denote SD (*n* = 4).

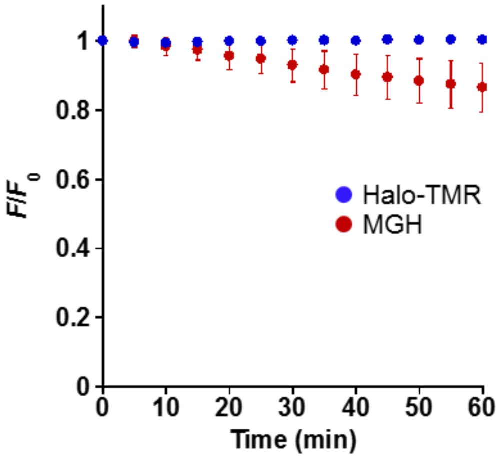

Fig. S7. Photostabilities of MGH and Halo-TMR during continuous irradiation (4.8 mW/cm^2). Changes in the fluorescence intensity (*F*) were normalized by the initial fluorescence intensity (F_0). The error bars denote SD (*n* = 3).

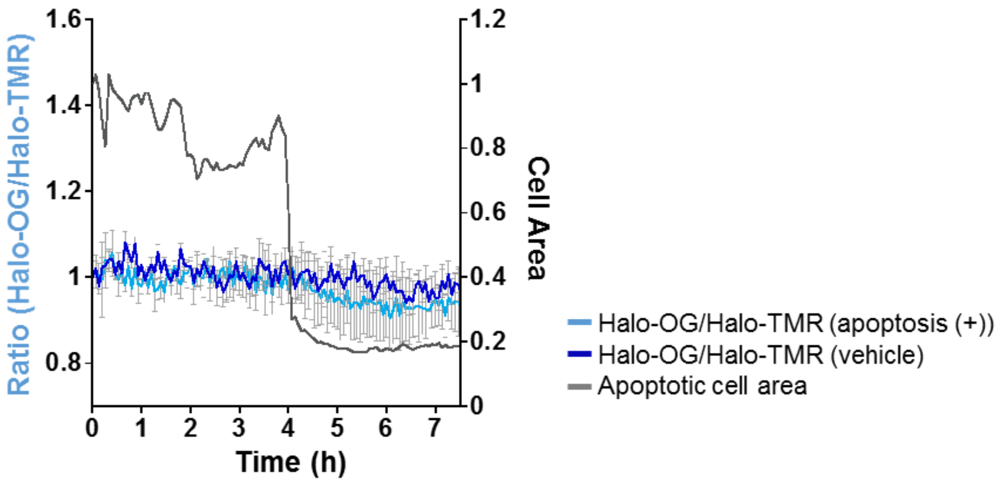

Fig. S8. Fluorescence ratio changes in Halo-OG/Halo-TMR with progression of apoptosis in HeLa cells. HeLa cells expressing HaloTag were loaded with 100 nM Halo-OG and 100 nM Halo-TMR for 30 min at 37 °C. Apoptosis inducers: anti-Fas antibody (250 ng/mL) and cycloheximide (10 µg/mL). The error bars denote SD (n = 3).

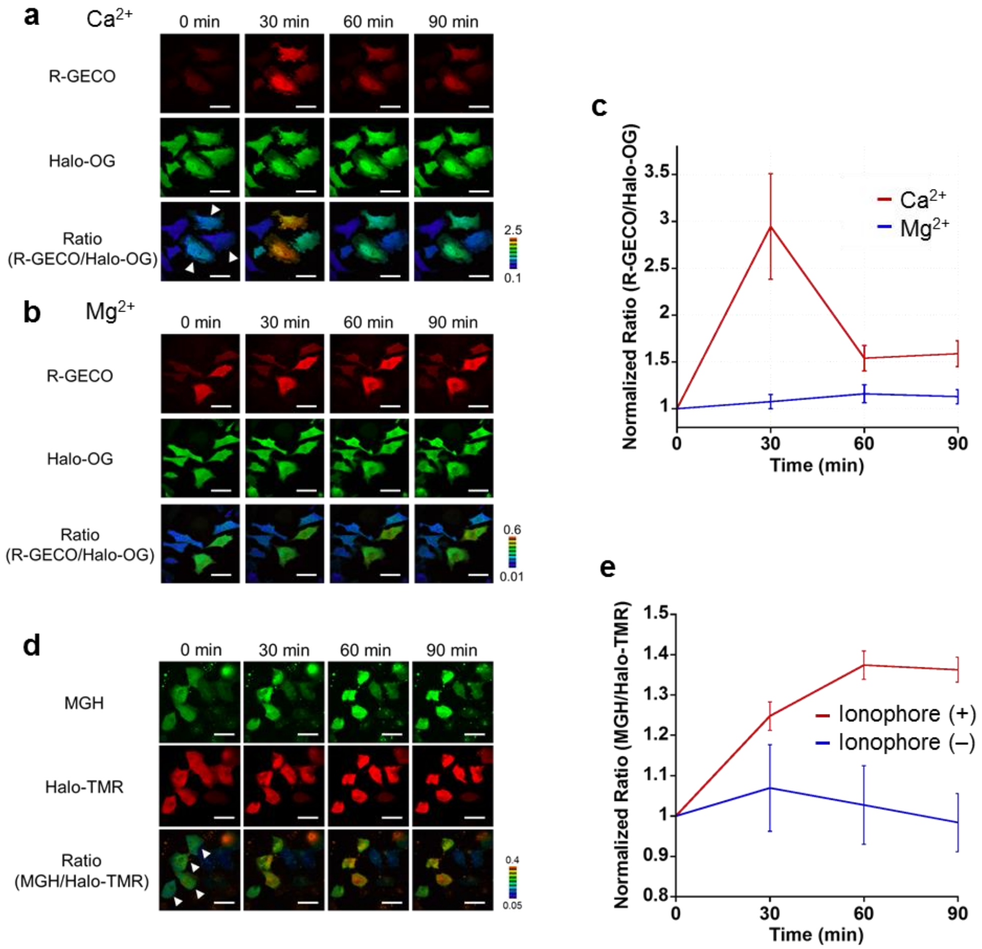

Fig. S9. Evaluation of the response of R-GECO to (a) Ca^{2+} and (b) Mg^{2+} in living HeLa cells. The intracellular concentrations of Mg^{2+} and Ca^{2+} were changed using an ionophore, 4-bromo-A23187. HeLa cells were transfected with plasmids encoding R-GECO and HaloTag, then HaloTag was labeled with 50 nM Halo-OG. The cells were treated with 2.5 µM 4-bromo-A23187 and (a) 100 µM Ca^{2+} or (b) 30 mM Mg^{2+}. The fluorescence of R-GECO was normalized to that of Halo-OG to exclude the influence of changes in probe concentrations during imaging. Scale bar: 40 µm. (c) The normalized fluorescence ratios of R-GECO/Halo-OG are presented as line plots. The error bars denote SD (n = 3). (d) Evaluation of the response of MGH to Mg^{2+} in living HeLa cells. The intracellular concentration of Mg^{2+} was changed using 4-bromo-A23187. HeLa cells were transfected with a plasmid encoding HaloTag. HaloTag was labeled with 3 µM MGH(AM) for 30 min and then 50 nM Halo-TMR for 15 min. The cells were treated with 2.5 µM 4-bromo-A23187 and 30 mM Mg^{2+}. The fluorescence of MGH was normalized to that of Halo-TMR to exclude the influence of changes in probe concentrations during imaging. Scale bar: 40 µm. (e) The normalized fluorescence ratios of MGH/Halo-TMR are presented as line plots. The error bars denote SD (n = 3).

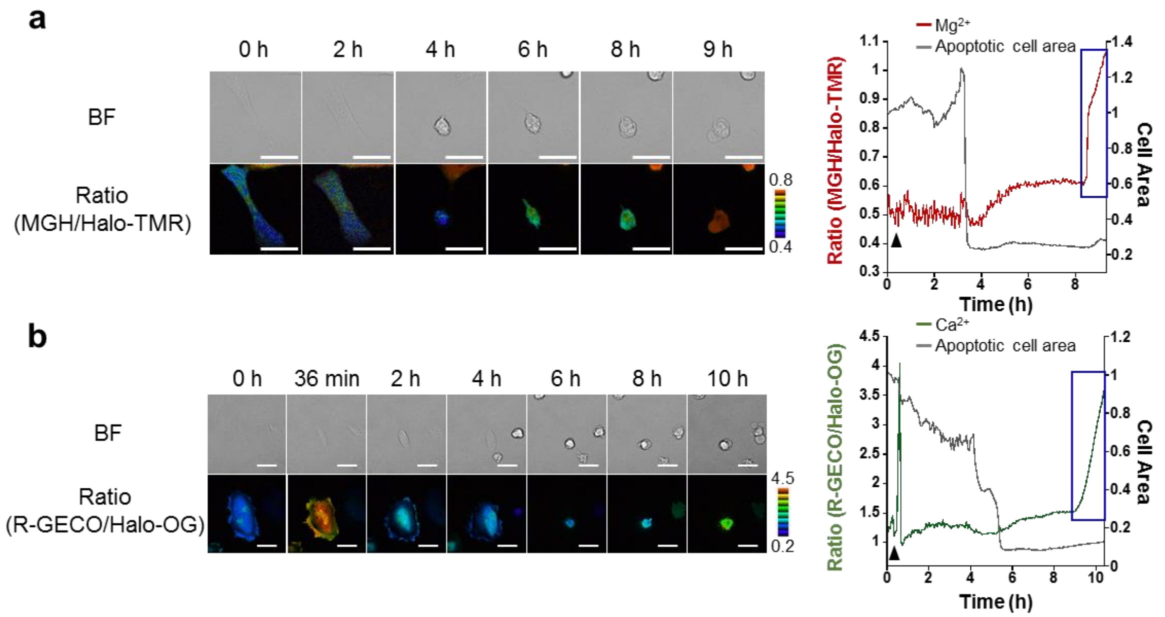

Fig. S10. Lack of integrity of the cell membrane during the late stages of apoptosis. Representative confocal fluorescence images of (a) Mg^{2+} or (b) Ca^{2+} dynamics with progression of apoptosis in HeLa cells. Anti-Fas antibody (250 ng/mL) and cycloheximide (10 µg/mL) as apoptosis inducers were added at the indicated time point (arrow). Scale bar: 40 µm.

2. Supporting Methods

Materials and instruments

All chemicals used for organic synthesis were of the best grade available, supplied by Tokyo Chemical Industries, Wako Pure Chemical, or Sigma-Aldrich Chemical Co., and were used as received without further purification. The pcDNA-3.1-(+) vector was purchased from Invitrogen (21083-027). Restriction endonucleases and PrimeSTAR® HS DNA polymerase were purchased from Takara Bio, Inc. Plasmid DNA was isolated using a QIAprep Spin Miniprep kit (Qiagen). MGH(AM) was dissolved in DMSO (biochemical grade, Wako) before fluorescence measurements to facilitate solubilization in aqueous solution. HaloTag TMR Ligand and HaloTag Oregon Green Ligand were purchased from Promega. Anti-Fas (CH11) antibody was purchased from MBL. Cycloheximide was purchased from Wako. CMV-R-GECO1 was purchased from Addgene. Annexin V (Alexa Fluor 350) and Annexin V (Alexa Fluor 680), Magnesium Green, Magnesium Green(AM), ER-Tracker Red and MitoTracker Deep Red FM were purchased from Thermo Fisher Scientific. 2-deoxyglucose was purchased from Wako Pure Chemical. KCN was purchased from Tokyo Chemical Industries. 4-bromo-A23187 was purchased from Sigma-Aldrich.

GPC purifications were performed with a JAIGEL 1H-2H column (Japan Analytical Industry Co., Ltd.) using a GPC system that was comprised of a pump (LC-6AD, Shimadzu) and a detector (SPD-20A, Shimadzu). HPLC analyses were performed with an Inertsil ODS-3 (4.6 mm×250 mm) column (GL Sciences Inc.) by using an HPLC system that was comprised of a pump (PU-2080, Jasco) and a detector (MD-2010 or FP-2020, Jasco). Preparative HPLC was performed with an Inertsil ODS-3 (10.0 mm × 250 mm) column (GL Sciences Inc.) using an HPLC system that was comprised of a pump (PU-2087, Jasco) and a detector (UV-2075, Jasco). Buffer A was composed of 0.1% HCOOH in H_2O (for MGH(AM)) or 50 mM triethylammonium acetate in H_2O (for MGH); Buffer B was composed of 0.1% HCOOH in acetonitrile (for MGH(AM)) or pure acetonitrile (for MGH). NMR spectra were recorded on a JEOL JNM-AL400 instrument at 400 MHz for ^{1}H and at 100 MHz for ^{13}C NMR or a Bruker Avance 500 instrument at 500 MHz for ^{1}H NMR and 125 MHz for ^{13}C NMR, using tetramethylsilane as an internal standard. Mass spectra were measured either on a Waters LCT-Premier XE or on a JMS-700 (JEOL) mass spectrometer.

Fluorescence spectra were measured by using a Hitachi F7000 spectrometer. The slit widths were 2.5 nm for both excitation and emission, and the photomultiplier voltage was 700 V. UV-visible absorption spectra were measured using a Jasco aV-650 spectrophotometer.

For photostability analysis of MGH and Halo-TMR, light irradiation was performed using a Xe light source (MAX-303; Asahi Spectra) equipped with band pass filters (490/5 nm for MGH; 550/5 nm for Halo-TMR).

The fluorescence microscopic images were recorded using a confocal fluorescence microscopic imaging system including a fluorescence microscope (IX71, Olympus), an EMCCD (iXon3, Andor Technology), a confocal scanner unit (CSU-X1, Yokogawa Electric Corporation), and a multispectral LED light source (Spectra X light engine, Lumencor). The filter sets were BP377 ± 25/DM405/BA447 ± 30 (for Hoechst 33342, Alexa Fluor350), BP438 ± 12/DM442/BA482 ± 17 and BA562 ± 20 (for ATeam), BP488 ± 3/DM488/BA520 ± 17.5 (for MGH, Magnesium Green and Halo-OG), BP560 ± 13/DM561/BA624 ± 20 (for ER-Tracker™ Red, R-GECO1.0, Halo-TMR) and BP640 ± 7/DM647/BA692 ± 20 (for MitoTracker® Deep Red FM and Alexa Fluor680). The entire system was controlled by using the MetaMorph 7.6 software (Molecular Devices).

Fluorometric analysis

The relative fluorescence quantum yields of the compounds were obtained by comparing the area under the emission spectrum. The following equation was used to calculate the quantum yield:

$$\Phi_x = \Phi_{st}\,(I_x/I_{st})(A_{st}/A_x)(n_x^2/n_{st}^2)$$

, where Φ_{st} is the reported quantum yield of the standard, I is the integrated emission spectrum, A is the absorbance at the excitation wavelength, and n is the refractive index of the solvent. The subscripts x and st denote the sample and the standard, respectively. Fluorescein (Φ = 0.85 when excited at 492 nm in 100 mM NaOH aq.) was used as the standard.

The photostabilities of MGH and Halo-TMR (1 µM, 2 mL) were examined in 100 mM HEPES buffer (pH 7.4) with 115 mM KCl and 20 mM NaCl at 25 °C under continuous irradiation through band pass filters (490 ± 2.5 nm for MGH, 550 ± 2.5 nm for Halo-TMR, 4.8 mW/cm^2) using a Xe light source. The fluorescence intensities of MGH (λ_{ex} = 515 nm, λ_{em} = 538 nm) and Halo-TMR (λ_{ex} = 555 nm, λ_{em} = 579 nm) were measured every 5 min for 1 h.

Determination of dissociation constants

The apparent dissociation constants (K_d) of MGH, HaloTag-MGH, and Magnesium Green for Mg^{2+} and Ca^{2+} in 100 mM HEPES buffer (pH 7.4) including 115 mM KCl and 20

mM NaCl at 37 °C were calculated using the following equation,

$$[M^{2+}] = K_d\,(F - F_{min})/(F_{max} - F)$$

where F is the fluorescence intensity at each metal ion concentration, F_{min} is the fluorescence intensity before addition of the metal ions, and F_{max} is the fluorescence intensity at the saturation state.

Metal ion selectivity study

Metal ion selectivity was measured by adding either $MgCl_2$, $CaCl_2$, $ZnCl_2$, $CoCl_2$, $MnCl_2$, $NiCl_2$ or $CuCl_2$ in 100 mM HEPES buffer (pH 7.4) with 115 mM KCl, 20 mM NaCl and 1 µM MGH at 37 °C.

Construction of plasmids

pcDNA-3.1-(+)-HaloTag

The DNA fragment of HaloTag was amplified from pFC14A-HaloTag, which was purchased from Promega, by PCR using primers (forward primer: 5'-AAAGACGCTAGCGCCGCCATGGGATCCGAAATCGGTACTG-3', reverse primer: 5'-ATAGCAAAGCTTACCGGAAATCTCCAGAGT-3'). The fragment was cleaved using *Nhe*I and *Hind*III, then ligated to *Nhe*I-*Hind*III site of pcDNA-3.1-(+)-BL-tag[S1] treated with the same restriction enzymes to generate pcDNA-3.1-(+)-HaloTag.

pcDNA-3.1-(+)-Halo-NLS

The DNA fragment of HaloTag was amplified from pFC14A-HaloTag by PCR using primers (forward primer: 5'-AAAGACGCTAGCGCCGCCATGGGATCCGAAATCGGTA CTG-3', reverse primer: 5'-GCGACTAAGCTTACCGGAAATCTCCAGAGTAGAC-3'). The fragment was cleaved using *Nhe*I and *Hind*III, then ligated to *Nhe*I-*Hind*III site of pcDNA-3.1-(+)-BL-NLS[S1] treated with the same restriction enzymes to generate pcDNA-3.1-(+)-Halo-NLS.

pcDNA-3.1-(+)-Lyn_{11}-Halo

The DNA fragment of HaloTag was amplified from pFC14A-HaloTag by PCR using primers (forward primer: 5'-AAAGACGGATCCGCCGCCATGGGATCCGAAATCGGTA CTG-3', reverse primer: 5'-ATAGCAGAATTCACCGGAAATCTCCAGAGTAGAC-3'). The fragment was cleaved using *Bam*HI and *Eco*RI, then ligated to *Bam*HI- *Eco*RI site of

pcDNA-3.1-(+) vector. The constructed plasmid was digested with *Hind*III and *Bam*HI and ligated in-frame into a similarly digested Lyn_{11} oligo DNA. The Lyn_{11} oligo DNA was amplified from a purchased oligo nucleotide template (5'-AATTAAAAGCTTGCCGCCATGGGATGTATAAAATCAAAAGGGAAAGACAGCGCGGGAGCAGATAGTGCTGGTAGTGCTGGTAGTGCTGGTGGATCCATCGGA-3' and 5'-TCCGATGGATCCACCAGCACTACCAGCACTACCAGCACTATCTGCTCCCGCGCTGTCTTTCCCTTTTGATTTTATACATCCCATGGCGGCAAGCTTTTAATT-3'; Gene Design, Inc.) using the following primers (forward primer: 5'-AATTAAAAGCTTGCCGC-3', reverse primer: 5'-AGGCTAGGATCCACCAG-3').

pKmcl-2xCOX8-Halo

The DNA fragment of HaloTag was amplified from pFC14A-HaloTag by PCR using primers (forward primer: 5'-GATAACGGATCCATGGGCTCCGAAATCG-3', reverse primer: 5'-AAGATCGAATTCTTAACCGGAAATCTCCAGAGTAGAC-3'). The fragment was cleaved using *Bam*HI and *Eco*RI, then ligated to pKmc-2xCOX8-BL, which was a kind gift from Dr. Atsushi Miyawaki, treated with the same restriction enzymes to generate pKmc-2xCOX8-Halo.

pmKate2-Halo-ER

The DNA fragment of HaloTag was amplified from pFC14A-HaloTag by PCR using primers (forward primer: 5'-GATAACACCGGTCGCCACCATGGGCTCCGAAATCG-3', reverse primer: 5'-AAGATCAGATCTACCTCCTCCACCACCGGAAATCTCCAGAGTAGAC-3'). The fragment was cleaved using *Age*I and *Bg*II, then ligated to pmKate2-ER, which was purchased from Evrogen (FP324), treated with the same restriction enzymes to generate pmKate2-Halo-ER.

pET21b(+)-His-Halo

The DNA fragment of HaloTag was amplified from pFC14A-HaloTag by PCR using primers (forward primer: 5'-GATAACGGATCCGGAAGGATTTCACATATGGGCTCCGAAATCGGTACTGG-3', reverse primer: 5'-AAGATCAAGCTTCTAACCGGAAATCTCCAGAGTAG-3'). The fragment was cleaved using *Bam*HI and *Hin*dIII then ligated to pET21b(+)-PYP3R[S2] treated with the same restriction enzymes to generate pET21b(+)-His-Halo.

Preparation of HaloTag protein

Hexahistidine-tagged HaloTag was overexpressed in Escherichia coli cells, BL21 (DE3), then the cells were cultivated in Luria-Bertani medium at 37 °C. When the OD_{600} of the culture medium reached 0.6–0.8, the culture flask was incubated at 20 °C and isopropyl-β-D-thiogalactopyranoside (final concentration: 100 μM) was added to the medium. After protein expression was induced overnight, the cells were collected by centrifugation at 4,700 ×*g* for 12 min, and were resuspended in 50 mM sodium phosphate buffer (pH 8.0) with 300 mM NaCl. After cell lysis by sonication, the lysate was centrifuged at 32,000 ×*g* for 20 min. The supernatant was loaded on cOmplete His-Tag Purification Resin. After the resin was washed with 50 mM sodium phosphate buffer (pH 8.0) with 300 mM NaCl and 5 mM imidazole, proteins adsorbed on the resin were eluted using 50 mM sodium phosphate buffer (pH 8.0) with 300 mM NaCl and 250 mM imidazole. Further purification was conducted by size exclusion chromatography (Superdex TM 75 10/300 GL, GE healthcare) using 100 mM HEPES buffer (pH 7.4) with 1 mM DTT to prevent dimerization of HaloTag. The purified protein was analyzed by SDS-PAGE for the purity check.

Detection of protein labeling by SDS-PAGE

HaloTag (40 μM) was added to a solution of MGH (30 μM) in 100 mM HEPES buffer (pH 7.4) with 115 mM KCl and 20 mM NaCl at 37 °C. After incubation for 1 h, the labeled protein was denatured in 2×SDS gel loading buffer (100 mM Tris-HCl buffer (pH 6.8), 4% SDS, 20% glycerol, and 10% mercaptoethanol) and resolved by SDS-PAGE. The fluorescence image of the gel was captured using a fluorescence image analyzer (Typhoon FLA 9500, GE Healthcare Bio-Sciences AB). The gels were stained with Coomassie Brilliant Blue prior to the capture of images.

Western blot analysis

HEK293 cells expressing CNNM4-FLAG in 24-well plates were lysed using 100 μL lysis buffer (100 mM Tris-HCl (pH 6.8), 4% SDS, 20% glycerol, and 100 mM DTT). The lysates were resolved by 10% SDS-PAGE and transferred onto polyvinylidene difluoride membranes. Anti-FLAG antibodies (1:1000) were used for detection of CNNM4-FLAG. Chemiluminescence was detected using the Amersham ECL Plus Western Blotting Detection System (GE Healthcare).

Cell culture

HEK293T cells, HEK293 cells, and HeLa cells were cultured in high-glucose Dulbecco's modified Eagle medium (DMEM) plus Gluta Max-I supplemented with 10% fetal bovine serum (FBS), 100 U/mL penicillin, and 100 µg/mL streptomycin. The cells were incubated at 37 °C in a humidified atmosphere of with 5% CO_2. A subculture was performed every 2–3 days from subconfluent (<80%) cultures using a trypsin-ethylenediamine tetraacetic acid solution. Transfection of plasmids was carried out in a glass-bottomed dish using Lipofectamine 3000 or 2000 according to the standard protocol.

Metal ion responsivity of MGH and R-GECO in HeLa cells

(R-GECO) HeLa cells were transfected with plasmids encoding R-GECO and HaloTag using Lipofectamine 3000. After 24 h, the cells were washed three times with HBSS and incubated in FBS-free DMEM containing 50 nM Halo-OG for 30 min at 37 °C under 5% CO_2. After washing with HBSS (free Ca^{2+} and Mg^{2+}), the cells were bathed in HBSS (free Ca^{2+} and Mg^{2+}). The cells were then treated with 2.5 µM 4-bromo-A23187 and 100 µM Ca^{2+} or 30 mM Mg^{2+}. The fluorescence of R-GECO was normalized according to the fluorescence of Halo-OG to exclude the influence of changes in the probe concentration and the fluctuations in the light intensity.

(MGH) HeLa cells were transfected with a plasmid encoding HaloTag using Lipofectamine 3000. After 24 h, the cells were washed three times with HBSS, incubated in FBS-free DMEM containing 3 µM MGH(AM) for 30 min, and then treated with 50 nM Halo-TMR for 15 min at 37 °C under 5% CO_2. After washing with HBSS (free Ca^{2+} and Mg^{2+}), the cells were bathed in HBSS (free Ca^{2+} and Mg^{2+}). The cells were then treated with 2.5 µM 4-bromo-A23187 and 30 mM Mg^{2+}. The fluorescence of MGH was normalized according to the fluorescence of Halo-TMR to exclude the influence of changes in the probe concentration and fluctuations in the light intensity.

Chemical synthesis

Scheme S1. Synthetic scheme for 5-amino APTRA (AM)

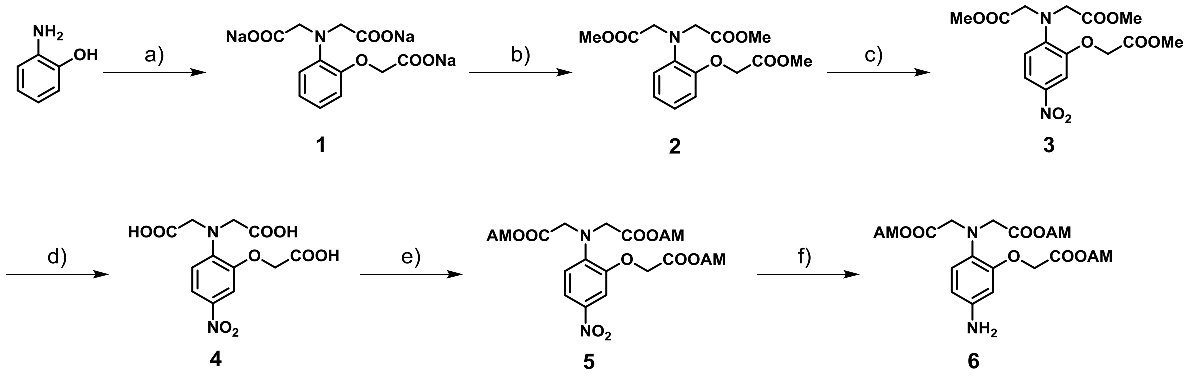

a) $ClCH_2COOH$, 2 M NaOH aq. 100 °C; b) H_2SO_4, MeOH, reflux, 31% (2 steps); c) HNO_3, AcOH, 0 °C, 77%; d) 2 M NaOH aq., MeOH/H_2O (3:1); e) bromomethyl acetate, TEA, DMF, r.t. 43% (2 steps); f) Pd/C, H_2, MeOH, r.t. quant.

Scheme S2. Synthetic scheme for alkynyl fluorescein derivative

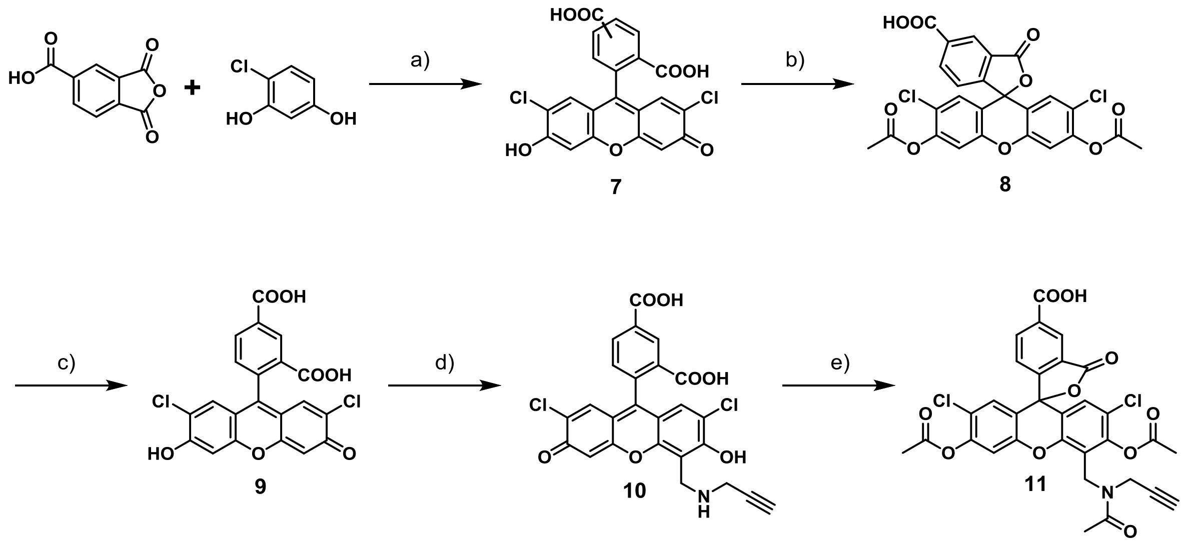

a) $MeSO_3H$, 90 °C; b) acetic anhydride, pyridine, reflux, 49% (2 steps); c) 2 M NaOH aq., MeOH/H_2O (3:1), r.t. quant.; d) propargylamine, formaldehyde, CH_3CN/H_2O (1:1), 80 °C; e) acetic anhydride, pyridine, reflux, 30% (2 steps).

Scheme S3. Synthetic scheme for HaloTag ligand

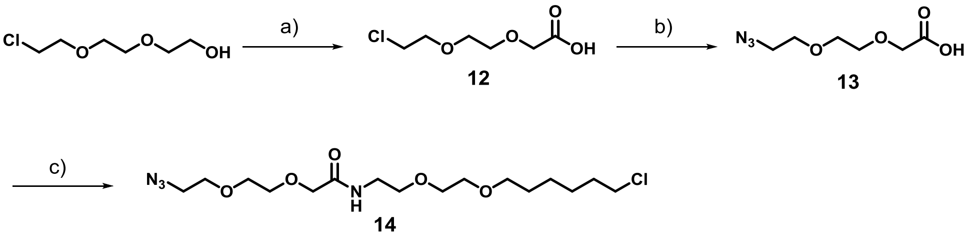

a) Cr_3O, 1.5 M H_2SO_4, acetone, 0 °C→r.t., 42%; b) NaN_3, H_2O, 80 °C, 68%; c) HaloTag Amine (O2) ligand, PyBOP, TEA, DMF, r.t., 32%.

Scheme S4. Synthetic scheme for MGH(AM) and MGH

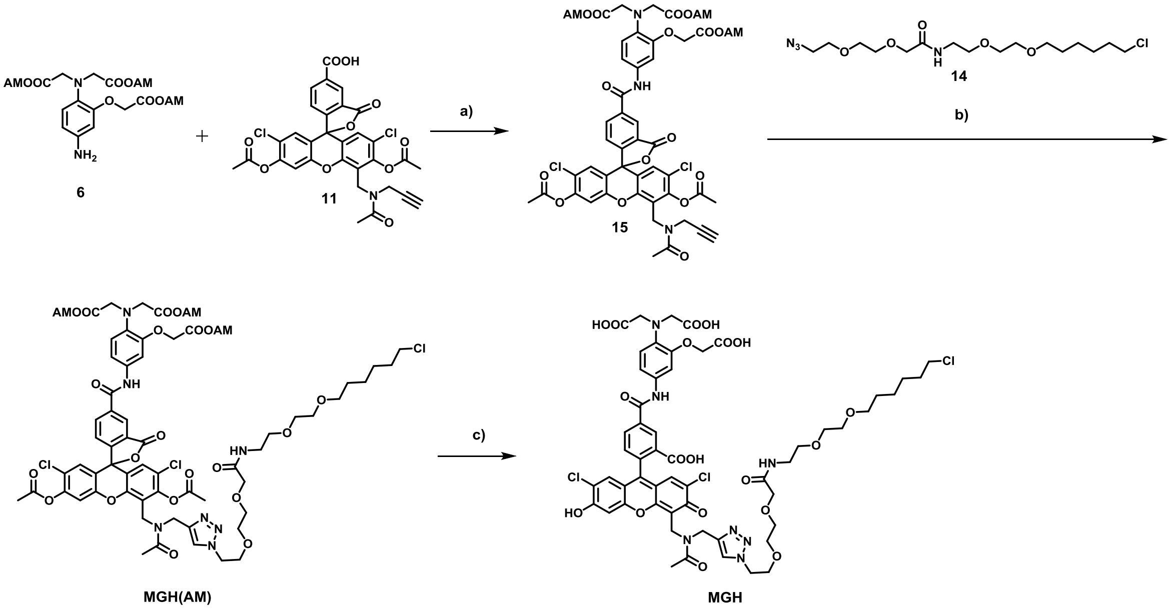

a) EEDQ, DCM, r.t., 29%; b) $CuSO_4$, sodium ascorbate, DMF/H_2O (4:1), r.t., 33%; c) 2 M NaOH aq., MeOH/H_2O (3:1), r.t., quant.

Synthesis of compounds

Compounds **1**–**3** were prepared according to the previously described procedures.[S3]

Synthesis of 1

o-Aminophenol (4.85 g, 44.5 mmol) and chloroacetic acid (21.0 g, 222 mmol) were added to a three-necked flask, and 2 M NaOH aq. (100 mL) was added to the solution until the pH decreased below 10. The mixture was stirred for 2 h at 100 °C. After cooling, the solvent was evaporated under reduced pressure. The crude mixture of compound **1**, excess NaOH and acetate residues was used in further synthesis without purification.
^{1}H NMR (400 MHz, D_2O) δ 6.69-6.79 (m, 4H), 4.35 (s, 2H), 3.73 (s, 4H).

Synthesis of 2
MeOH (120 mL) and H_2SO_4 (8.60 mL, 342 mmol) were added to the crude residue obtained in the preparation of compound **1**, and the mixture was stirred for 3 days at reflux temperature. After cooling, the salts were filtered off and the solvent was evaporated. The residue was dissolved in ethyl acetate and washed with 2 M NaOH aq. and then with brine. After the organic layer was dried over Na_2SO_4 and evaporated under reduced pressure, the residue was purified by flash column chromatography on silica gel (ethyl acetate/hexane = 3:7). Compound **2** (2.11 g, 31%) was obtained as a brown oil.
^{1}H NMR (400 MHz, $CDCl_3$) δ 6.79–6.95 (m, 4H), 4.66 (s, 2H), 4.21 (s, 4H), 3.78 (s, 3H), 3.72 (s, 6H); MS (ESI^{+}): Calcd for [M+H]$^{+}$ 326.1162, found 326.0683.

Synthesis of 3
Compound **2** (837 mg, 2.57 mmol) was dissolved in AcOH (10 mL), and fuming HNO_3 (160 µL, 3.86 mmol) dissolved in AcOH (1.0 mL) was added in a dropwise manner at 0 °C for over 5 min. After confirming the completion of the reaction, the reaction mixture was poured into ice-water. After extraction with DCM, the organic layer dried over with Na_2SO_4 and filtered. After removing of the solvent under reduced pressure, the residue was purified by column chromatography on silica gel (DCM/MeOH = 99.5:0.5). Compound **3** (738 mg, 77%) was obtained as a yellow solid.
^{1}H NMR (400 MHz, $CDCl_3$) δ 3.78 (s, 6H), 3.82 (s, 3H), 4.28 (s, 4H), 4.68 (s, 2H), 6.73 (d, *J* = 8.8 Hz, 1H), 7.62 (d, *J* = 2.0 Hz, 1H), 7.86 (dd, *J* = 2.0, 8.8 Hz, 1H); MS (ESI^{+}): Calcd for [M+H]$^{+}$ 371.1012, found 371.5169.

Synthesis of 4
Compound **3** (700 mg) was dissolved in 20 mL of MeOH/H_2O (3:1) and 3 mL of 2 M NaOH aqueous solution was added dropwise at 0 °C. The reaction mixture was then warmed to

room temperature. After stirring for 6 h, Dowex-50 H^+ resin was added into the reaction mixture and the pH was adjusted to 5–6, then was filtered off, and the solvent was removed under reduced pressure. Compound **4** (620 mg) was obtained as an orange powder.

Synthesis of 5

Compound **4** (620 mg, 1.88 mmol) was dissolved in dry DMF (10.0 mL). Bromomethyl acetate (1.41 mL, 15.0 mmol) and dry TEA (3.15 mL, 22.6mmol) were added at room temperature under Ar. After stirring for 1 day, the solvent was removed under reduced pressure, and DCM was added to the residue, and washed with water. The organic layer was washed with brine, dried with Na_2SO_4 and evaporated. The residue was purified by GPC. Compound **5** (439 mg, 43%) was obtained as a yellow oil.

^{1}H NMR (500 MHz, $CDCl_3$) δ 7.87 (dd, *J* = 9.0, 2.5 Hz, 1H), 7.61 (d, *J* = 2.5 Hz, 1H), 6.74 (d, *J* = 9.0 Hz, 1H), 5.84 (s, 2H), 5.82 (s, 4H), 4.75 (s, 2H), 4.30 (s, 4H), 2.13 (s, 9H); ^{13}C NMR (125 MHz, $CDCl_3$); δ 169.5, 169.2, 166.5, 147.5, 144.9, 141.3, 119.2, 116.9, 108.7, 79.8, 79.4, 65.3, 53.8, 20.7, 20.6; HRMS (FAB^+): Calcd for $[M+H]^+$ 545.1770, found 545.1260.

Synthesis of 6

Compound **5** (200 mg, 0.37 mmol) was dissolved in MeOH (20 ml). Pd/C (20%, 18.8 mg) was added and the reaction was stirred for 1 h under H_2. The solution was filtered through a layer celite and evaporated under reduced pressure. Compound **6** (190 mg, quant.) was obtained as a yellow oil.

^{1}H NMR (400 MHz, $CDCl_3$) δ 6.87 (d, *J* = 8.0 Hz), 6.27 (dd, *J* = 8.0, 2.4 Hz, 1H), 6.21 (d, *J* = 2.4 Hz, 1H), 5.82 (s, 2H), 5.74 (s, 4H), 4.70 (s, 2H), 4.13 (s, 4H), 2.11 (s, 3H), 2.08 (s, 6H); ^{13}C NMR (100 MHz, $CDCl_3$) δ 170.0, 169.5, 167.9, 151.7, 143.4, 130.7, 123.5, 109.2, 103.4, 79.3, 79.2, 66.0, 54.0, 20.6; MS (ESI^+): Calcd for $[M+H]^+$ 515.1435, found 514.9805.

Compounds **7**–**9** were prepared according to the previously described procedures.[S4]

Synthesis of 7

4-Chlororesorcinol (4.89 g, 33.8 mmol) and 4-carboxyphthalic anhydride (3.25 g, 16.9 mmol) were stirred in methanesulfonic acid (40 mL) at 90 °C for 14 h. The reaction mixture was then poured into 400 mL of stirred ice water, and the resulting suspension was filtered The residue was washed with H_2O and dried under vacuum at 90 °C overnight to give a

brown solid (7.58 g). The product was carried forward without further purification.

Synthesis of 8

The brown solid (7.58 g) was stirred in 25 mL of acetic anhydride and 1.5 mL of pyridine and heated to reflux for 30 min. The reaction mixture was cooled to room temperature for 4 h and then filtered. The filtrate was added slowly into 75 mL of stirred H_2O, and the mixture was stirred for additional 10 min, and then extracted with ethyl acetate. The combined organic layer was washed with 0.4 M HCl aq. and brine, dried with Na_2SO_4, and evaporated to give compound **8** (4.40 g, 49%) as a yellow solid.

^{1}H NMR (500 MHz, CD_3Cl) δ 8.79 (d, *J* = 1.0 Hz, 1H), 8.45 (dd, *J* = 8.0, 1.5 Hz, 1H), 7.33 (d, *J* = 8.0 Hz, 1H), 7.18 (s, 2H), 6.87 (s, 2H), 2.83 (s, 6H); MS (ESI^+): Calcd for $[M+H]^+$ 529.0015, found 529.0502.

Synthesis of 9

Compound **8** (2.20 g, 4.16 mmol) was dissolved in 24 mL of MeOH/H_2O (3:1). 2 M NaOH aq. (4.5 mL) was added dropwise at 0 °C. The color of the solution changed quickly from yellow to orange. The reaction mixture was warmed at room temperature. After stirring for 2 h, Dowex-50 H^+ resin was added into the reaction mixture and the pH was adjusted to 5–6, then was filtered off, and the solvent was removed under reduced pressure. Compound **9** (1.85 g, quant.) was obtained as an orange powder.

^{1}H NMR (500 MHz, DMSO-d_6) δ 11.35 (bs, 2H), 8.42 (s, 1H), 8.31 (d, *J* = 8.0 Hz, 1H), 7.43 (d, *J* = 8.0 Hz, 1H), 6.91 (s, 2H), 6.78 (s, 2H); MS (ESI^-): Calcd for $[M–H]^-$ 442.9804, found 442.9261.

Synthesis of 10

Propargylamine (218 µL, 4.04 mmol) was combined with 37% formaldehyde aqueous solution (253 µL, 3.37 mmol) in 13 mL of acetonitrile under Ar and heated to reflux for 1 h. Compound **9** (1.50 g, 3.37 mmol) dissolved in 28 mL acetonitrile and 28 mL H_2O was added to the reaction solution, and the mixture was refluxed for 3 h. The reaction mixture was cooled, and the solvent was removed under reduced pressure to give compound **10** (1.30 g) as a red solid. The product was carried forward without further purification.

MS (ESI^+): Calcd for $[M+H]^+$ 512.0226, found 511.9580.

Synthesis of 11

Compound **10** (1.30 g) was stirred in 20 mL of acetic anhydride, and 800 µL of pyridine, and then heated to reflux for 1 h. After cooling at room temperature for 2 h, the reaction mixture was added slowly to 450 mL of stirred H_2O. After stirred for an additional 10 min, the mixture was extracted with ethyl acetate. The combined organic layer were washed with H_2O, 0.4 M HCl aq. and brine, dried with Na_2SO_4 and evaporated. The residue was purified by column chromatography on silica gel (DCM/MeOH = 99:1). Compound **11** (645 mg, 30%) was obtained as a colorless oil.

^{1}H NMR (500 MHz, $CDCl_3$) δ 8.80 (d, J = 1.0 Hz, 1H), 8.47 (dd, J = 8.0, 1.0 Hz, 1H), 7.35 (d, J = 8.0 Hz, 1H), 7.28 (s, 1H), 6.87 (s, 1H), 6.86 (s, 1H), 4.87–5.12 (m, 2H), 3.89 (d, J = 2.5 Hz, 2H), 2.40 (s, 3H), 2.38 (s, 3H), 2.31 (t, J = 2.5 Hz, 1H), 2.25 (s, 3H); ^{13}C NMR (125 MHz, $CDCl_3$) δ 171.4, 168.3, 168.0, 167.9, 167.4, 155.4, 149.3, 148.9, 148.8, 148.7, 137.2, 133.2, 128.6, 128.3, 127.9, 126.2, 124.3, 123.7, 123.3, 120.0, 117.1, 116.8, 113.3, 80.7, 78.0, 72.9, 37.0, 36.9, 21.5, 20.6, 20.5; HRMS (FAB$^+$): Calcd for $[M+H]^+$ 638.0543, found 638.0616.

Compounds **12** and **13** were prepared according to the previously described procedures.[S5]

Synthesis of 12

Chromium trioxide (25.0 g, 162 mmol) was dissolved in 300 mL of 1.5 M H_2SO_4 aq., and the solution was cooled to 0 °C. 2-[2-(chloroethoxy)ethoxy]ethanol (8.30 g, 49.2 mmol) in 150 mL of acetone was dropwisely added, and the reaction mixture was stirred at room temperature for 3 h. The solvent was removed under reduced pressure, and the aqueous layer was extracted with DCM. The combined organic layer was washed with brine and dried with Na_2SO_4 and evaporated. Compound **12** (4.89 g, 42%) was obtained as a colorless oil.

^{1}H NMR (500 MHz, $CDCl_3$) δ 10.21 (bs, 1H), 4.23 (s, 2H), 3.79 (t, J = 5.5 Hz, 4H), 3.73 (t, J = 5.5 Hz, 2H), 3.65 (t, J = 5.5 Hz, 2H); MS (ESI$^-$): Calcd for $[M–H]^-$ 181.0346, found 180.9873.

Synthesis of 13

Compound **12** (3.38 g, 18.5 mmol) and NaN_3 (4.81 g, 74.0 mmol) in 13 mL of H_2O were stirred with heating at 80 °C for 32 h. After cooling to room temperature, the reaction mixture was acidified with 2 M HCl aq. and extracted with DCM. The combined organic layer was dried with Na_2SO_4 and evaporated. Compound **13** (2.39 g, 68%) was obtained as a colorless

oil.

^{1}H NMR (500 MHz, $CDCl_3$) δ 9.40 (bs, 1H), 4.22 (s, 2H), 3.77–3.79 (m, 2H), 3.69–3.73 (m, 4H), 3.42 (t, *J* = 5.0 Hz, 2H); MS (ESI$^-$): Calcd for $[M–H]^-$ 188.0750, found 188.3099.

Synthesis of 14

Compound **13** (63.4 mg, 0.330 mmol) was dissolved in dry DMF (1 mL). PyBOP (229 mg, 0.44 mmol) and dry TEA (124 μL, 0.88 mmol) were added at room temperature under Ar. After stirring for 30 min, HaloTag® Amine (O2) Ligand (50.0 mg, 0.22 mmol) was added at room temperature. After stirring for 15 h, the solvent was removed under reduced pressure and ethyl acetate was added to the residue, and washed with 10% citric acid and water. The organic layer was washed with brine, dried with Na_2SO_4 and evaporated. The residue was purified by column chromatography on silica gel (DCM/MeOH = 98:2) and subsequently by GPC. Compound **14** (28.0 mg, 32%) was obtained as a colorless oil.

^{1}H NMR (500 MHz, $CDCl_3$) δ 4.01 (s, 2H), 3.67–3.70 (m, 6H), 3.61–3.63 (m, 2H), 3.55–3.59 (m, 4H), 3.46 (t, *J* = 6.5 Hz, 2H), 3.42 (t, *J* = 5.0 Hz, 2H), 1.75–1.81 (m, 2H), 1.58–1.63 (m, 2H), 1.30–1.34 (m, 4H); ^{13}C NMR (125 MHz, $CDCl_3$) δ 169.8, 71.3, 70.9, 70.6, 70.3, 70.1, 70.0, 69.8, 50.6, 45.1, 38.6, 32.5, 29.5, 26.7, 25.4; HRMS (FAB$^+$): Calcd for $[M+H]^+$ 395.1983, found 395.2067.

Synthesis of 15

Compound **11** (143 mg, 0.224 mmol) and EEDQ (60.9 mg, 0.246 mmol) were stirred in dry DCM (3 mL) at room temperature for 5 min under Ar. Compound **6** (139 mg, 0.269 mmol) was dissolved in dry DCM (2 mL) and added. The reaction was continued for 19 h, and the solution was evaporated. The residue was purified by column chromatography on silica gel (DCM/MeOH = 99:1). Compound **15** (73.7 mg, 29%) was obtained as a yellow solid.

^{1}H NMR (500 MHz, $CDCl_3$) δ 8.56 (s, 1H), 8.35 (d, *J* = 8.0 Hz, 1H), 8.20 (s, 1H), 7.37 (d, *J* = 8.0 Hz, 1H), 7.28 (s, 1H), 7.28 (s, 1H), 6.89 (d, *J* = 7.0 Hz, 1H), 6.89 (d, *J* = 7.0 Hz, 1H), 6.87 (s, 1H), 6.86 (s, 1H), 5.83 (s, 2H), 5.79 (s, 4H), 4.87–5.12 (m, 2H), 4.74 (s, 2H), 4.26 (s, 4H), 3.89 (d, *J* = 2.5 Hz, 2H), 2.38 (s, 3H),2.31 (s, 3H), 2.31 (t, *J* = 2.5 Hz, 1H), 2.24 (s, 3H), 2.15 (s, 3H), 2.12 (s, 6H); ^{13}C NMR (125 MHz, $CDCl_3$) δ 170.3,170.1, 170.0, 169.6, 168.2, 167.9, 167.5, 167.4, 163.2, 154.0, 149.8, 149.5, 149.0, 148.9, 148.8, 137.9, 135.9, 135.4, 133.0, 128.6, 128.2, 126.4, 124.8, 123.8, 123.7, 123.3, 120.7, 120.4, 116.9, 116.8, 114.4, 113.3, 107.7, 80.9, 79.7, 79.4, 78.3, 72.6, 65.7, 53.5, 36.8, 36.6, 21.7, 20.8, 20.7, 20.6,

20.5; HRMS (FAB^+): Calcd for M^+ 1133.1872, found 1133.1878.

Synthesis of MGH(AM)

Compound **15** (9.71 mg, 8.56 μmol) and compound **14** (4.06 mg, 10.3 μmol) were dissolved in 200 µL of DMF/H_2O (4:1). $CuSO_4$ (1.64 mg, 10.3 μmol) and sodium ascorbate (2.04 mg, 10.3 μmol) were added at room temperature. After stirring for 1.5 h, the solvent was removed under reduced pressure and ethyl acetate was added to the residue, and washed with water. The organic layer was washed with brine, dried with Na_2SO_4 and evaporated. The residue was purified by HPLC. MGH(AM) (4.32 mg, 33%) was obtained as a colorless powder.

^{1}H NMR (500 MHz, Acetone-d_6) δ 9.84 (s, 1H), 8.57 (d, J = 1.0 Hz, 1H), 8.42 (dd, J = 8.0, 1.0 Hz, 1H), 7.86 (s, 1H), 7.68 (d, J = 8.0 Hz, 1H), 7.60 (d, J = 2.5 Hz, 1H), 7.45 (dd, J = 9.0, 2.5 Hz, 1H), 7.40 (s, 1H), 7.13–7.17 (m, 3H), 6.97 (d, J = 9.0 Hz, 1H), 5.84 (s, 2H), 5.77 (s, 4H), 4.92–5.11 (m, 2H), 4.85 (s, 2H), 4.59 (t, J = 5.5 Hz, 2H), 4.55 (s, 2H), 4.28 (s, 4H), 3.92 (t, J = 5.5 Hz, 2H), 3.84 (s, 2H), 3.63–3.64 (m, 4H), 3.59 (t, J = 7.0 Hz, 2H), 3.48–3.56 (m, 6H), 3.41 (t, J = 8.0 Hz, 2H), 3.34–3.37 (m, 2H), 2.28–2.44 (m, 9H), 2.08 (s, 3H), 2.06 (s, 6H), 1.72–1.78 (m, 2H), 1.50–1.56 (m, 2H), 1.41–1.47 (m, 2H),1.34–1.39 (m, 2H); ^{13}C NMR (125 MHz, $CDCl_3$) δ 171.8, 171.5, 170.9, 169.7, 169.6, 169.5, 169.4, 169.1, 165.2, 155.9, 151.5, 151.3, 150.8, 150.6, 150.3, 145.5, 139.8, 137.4, 137.0, 135.8, 130.5, 129.6, 128.2, 126.4, 126.1, 124.9, 124.8, 124.4, 123.4, 121.8, 119.4, 119.3, 115.9, 115.3, 109.7, 82.1, 80.9, 80.8, 72.4, 72.3, 72.1, 71.8, 71.7, 71.2, 71.0, 67.2, 55.02, 51.5, 46.7, 44.1, 40.0, 38.3, 34.3, 28.3, 27.1, 23.0, 21.7, 21.5, 21.4; HRMS (FAB^+): Calcd for $[M]^+$ 1527.3855, found 1527.3840. HPLC chromatogram after purification is shown below. Elution was performed with a 30-minlinear gradient from 55% CH_3CN/0.1% HCOOH to 70% CH_3CN/0.1% HCOOH.

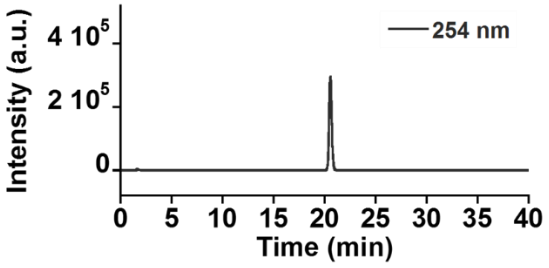

Synthesis of MGH

MGH(AM) (2.41 mg, 1.58 μmol) was dissolved in MeOH (7 mL). 2 M NaOH aq. (31.2 µL,

63.2 µmol) was added dropwise and stirred at 0 °C for 1 day. Reaction completion was checked by HPLC, then 100 mM HEPES buffer (pH 7.4) with 115 mM KCl and 20 mM NaCl was added to the reaction solution and diluted to a final concentration 3 mM. The stock solution was quickly frozen by liquid nitrogen, and stored below –20 °C.

MS (ESI$^-$): Calcd for $[M–H]^-$ 1226.3010, found 1226.0365. HPLC chromatogram after reaction is shown below. Elution was performed with a 15-minlinear gradient from 10% CH_3CN to 90% CH_3CN.

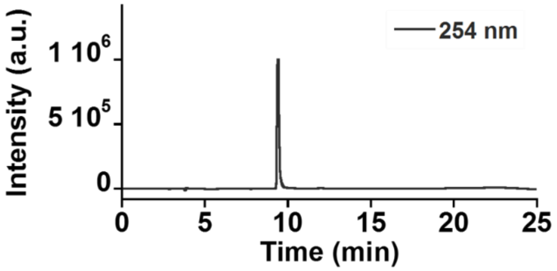

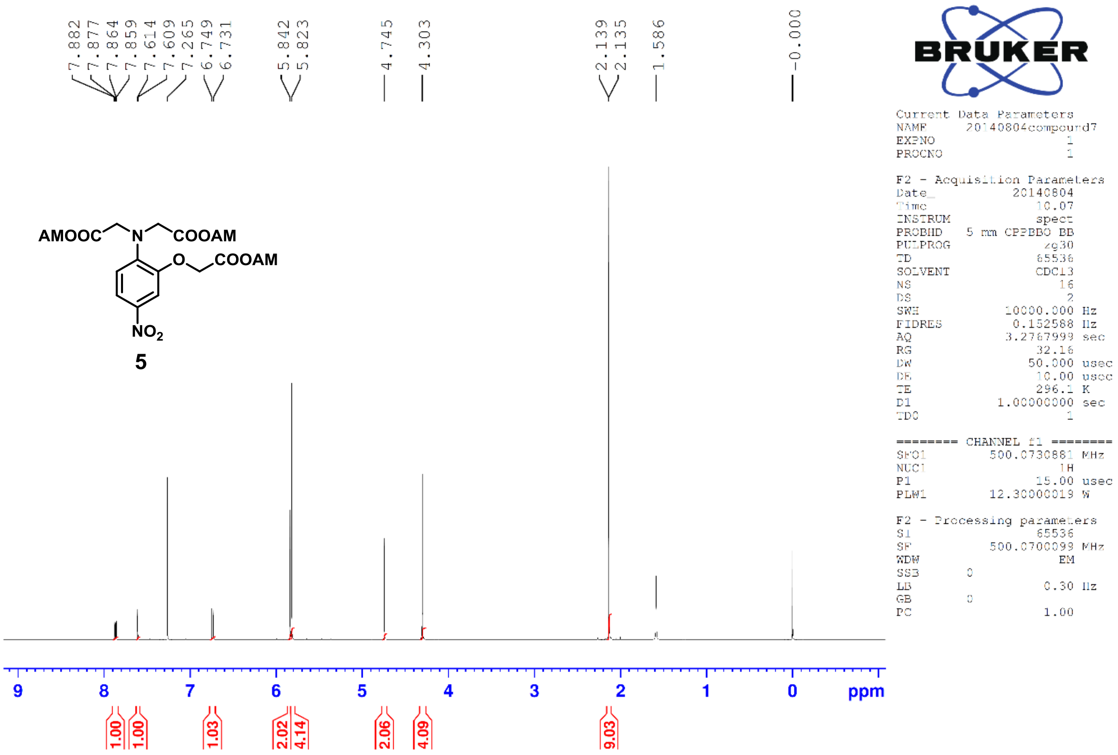

^{1}H NMR spectrum of compound **5**

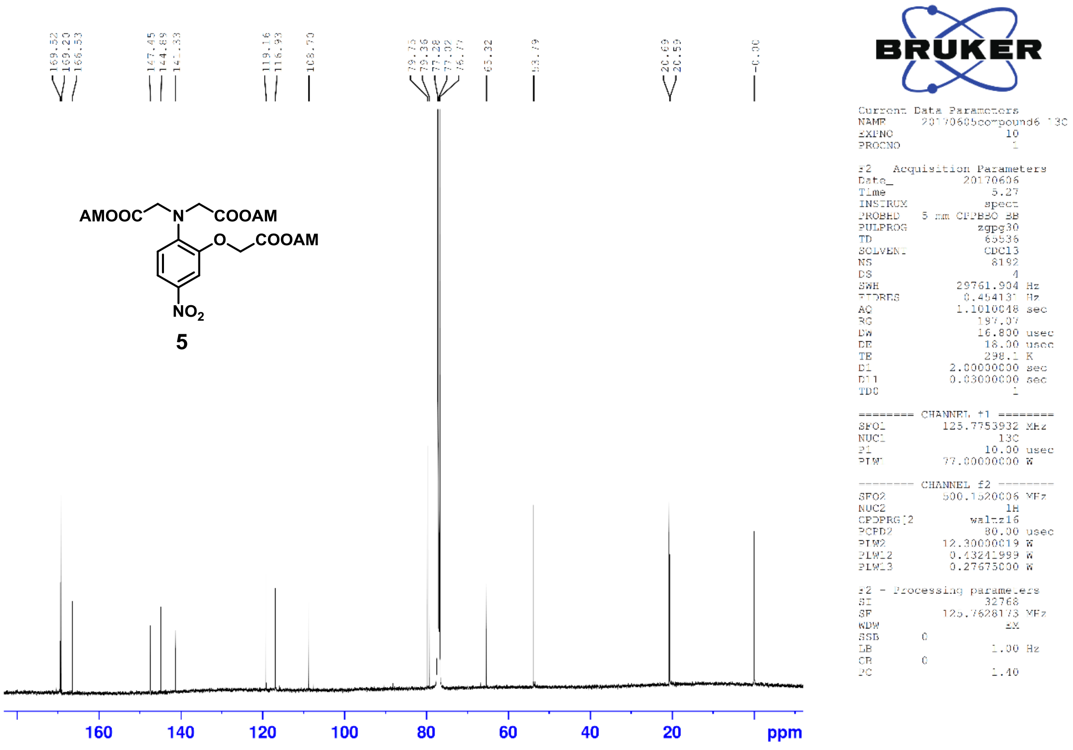

^{13}C NMR spectrum of compound **5**

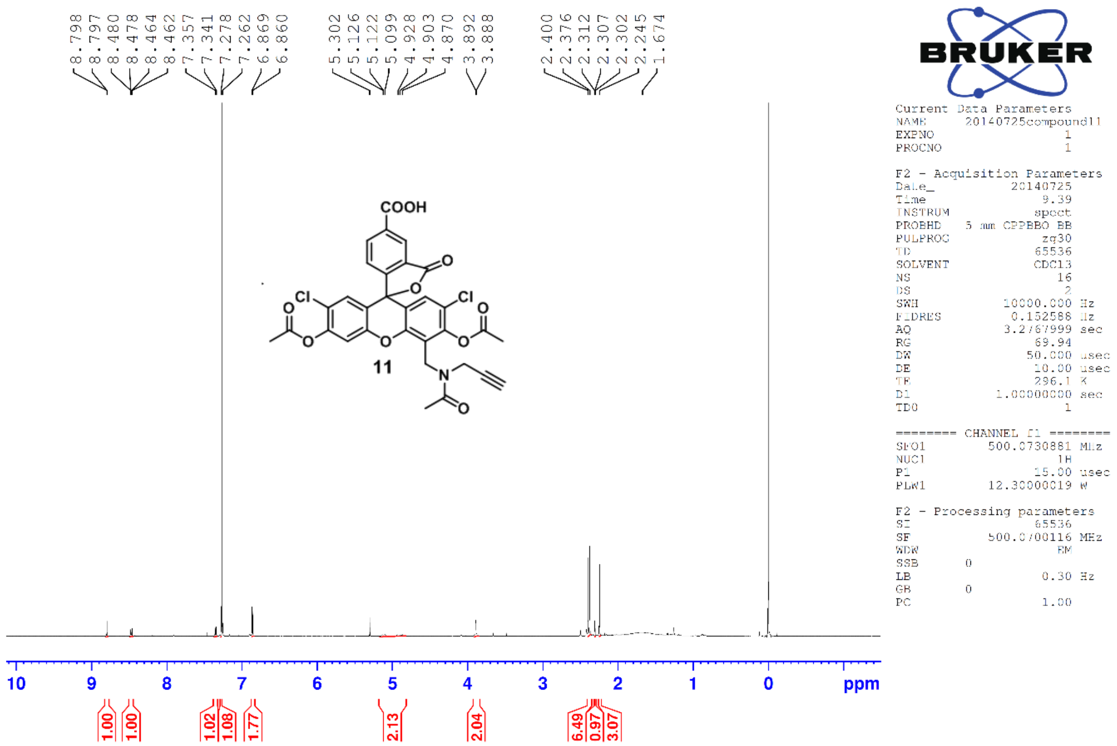

^{1}H NMR spectrum of compound **11**

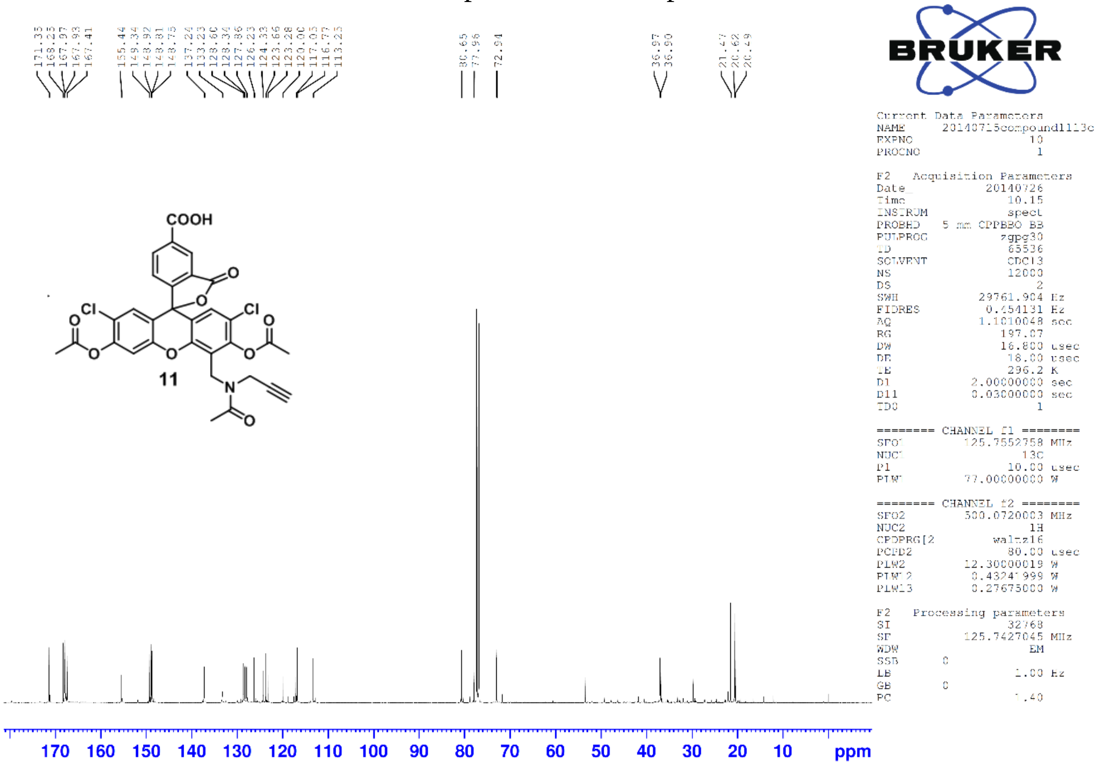

^{13}C NMR spectrum of compound **11**

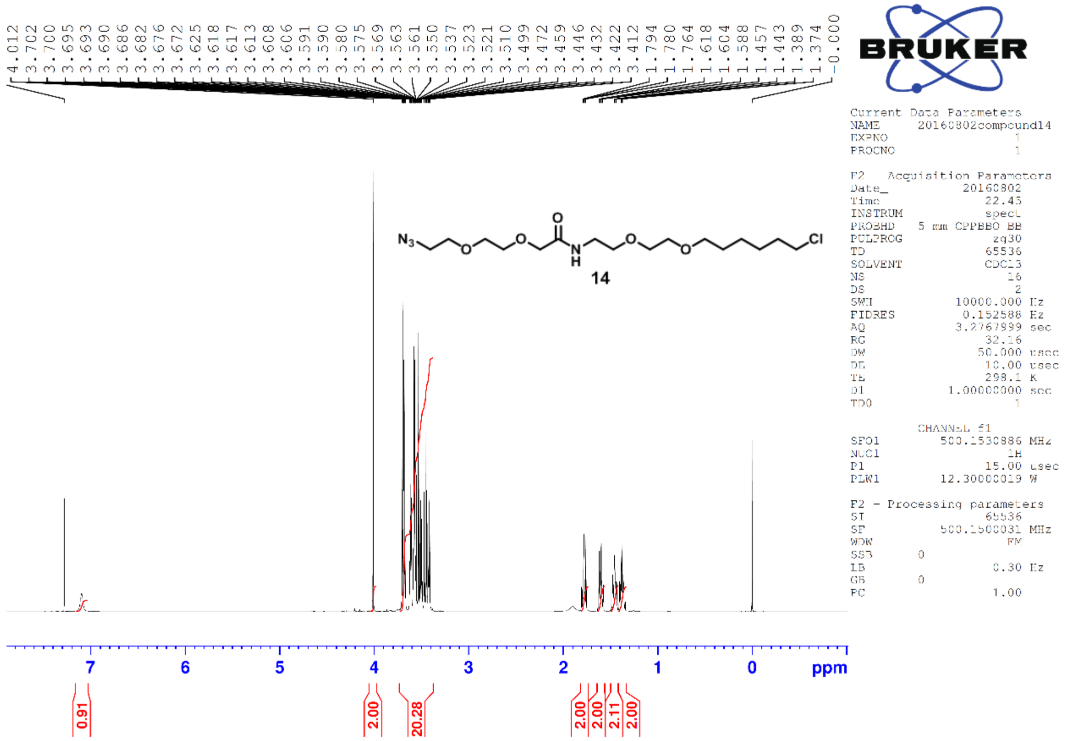

^{1}H NMR spectrum of compound **14**

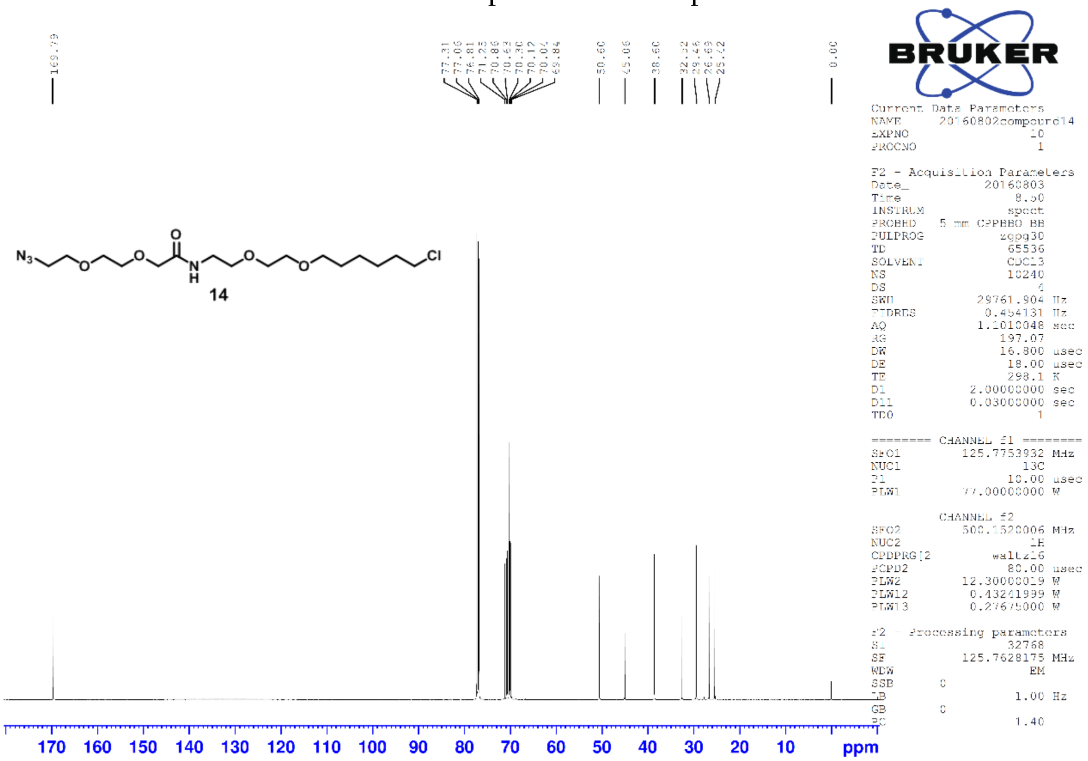

^{13}C NMR spectrum of compound **14**

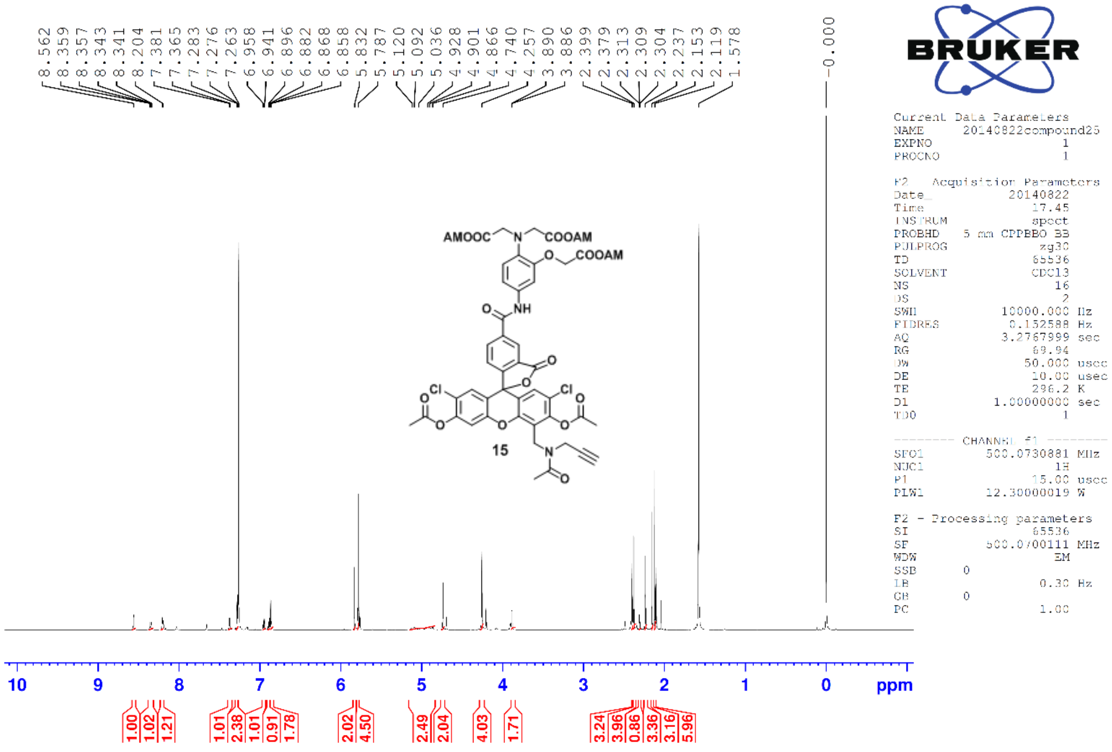

^{1}H NMR spectrum of compound **15**

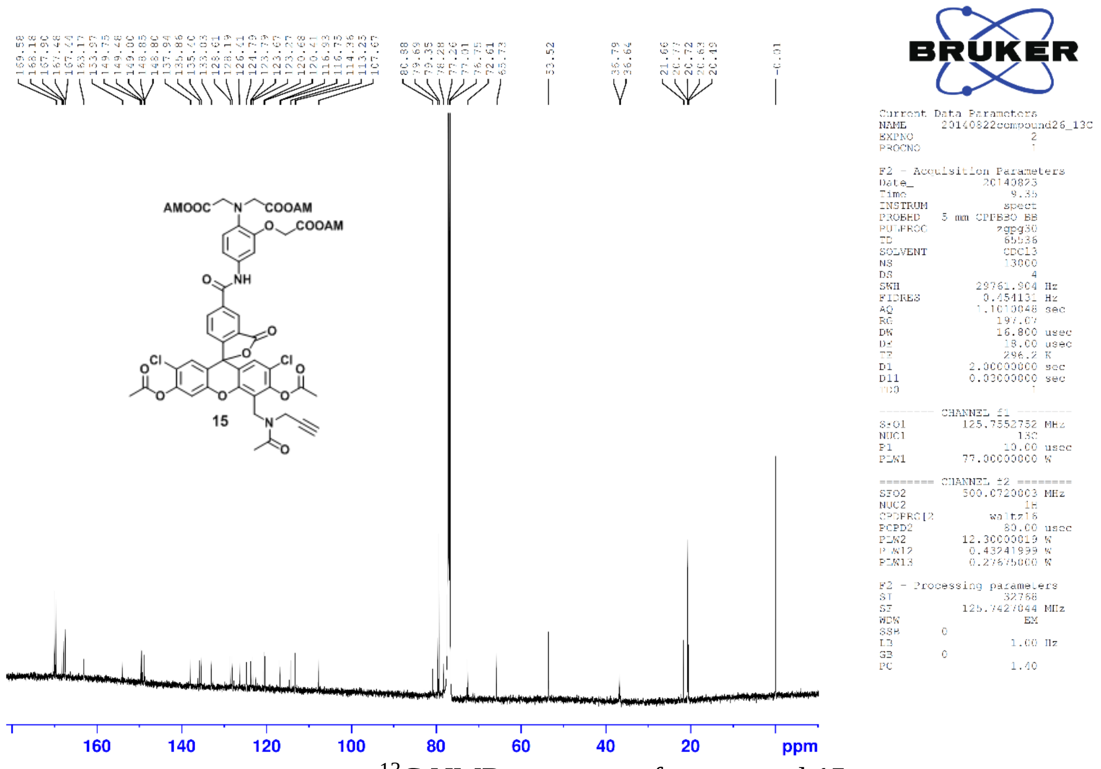

^{13}C NMR spectrum of compound **15**

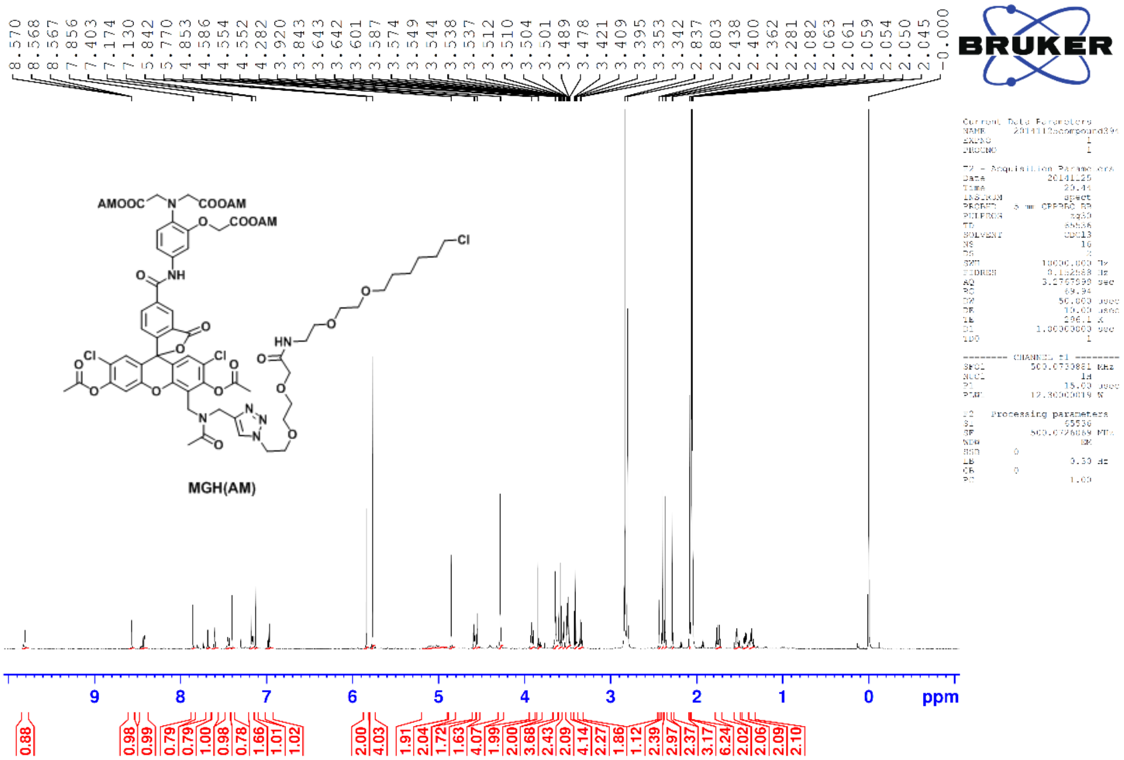

^{1}H NMR spectrum of MGH(AM)

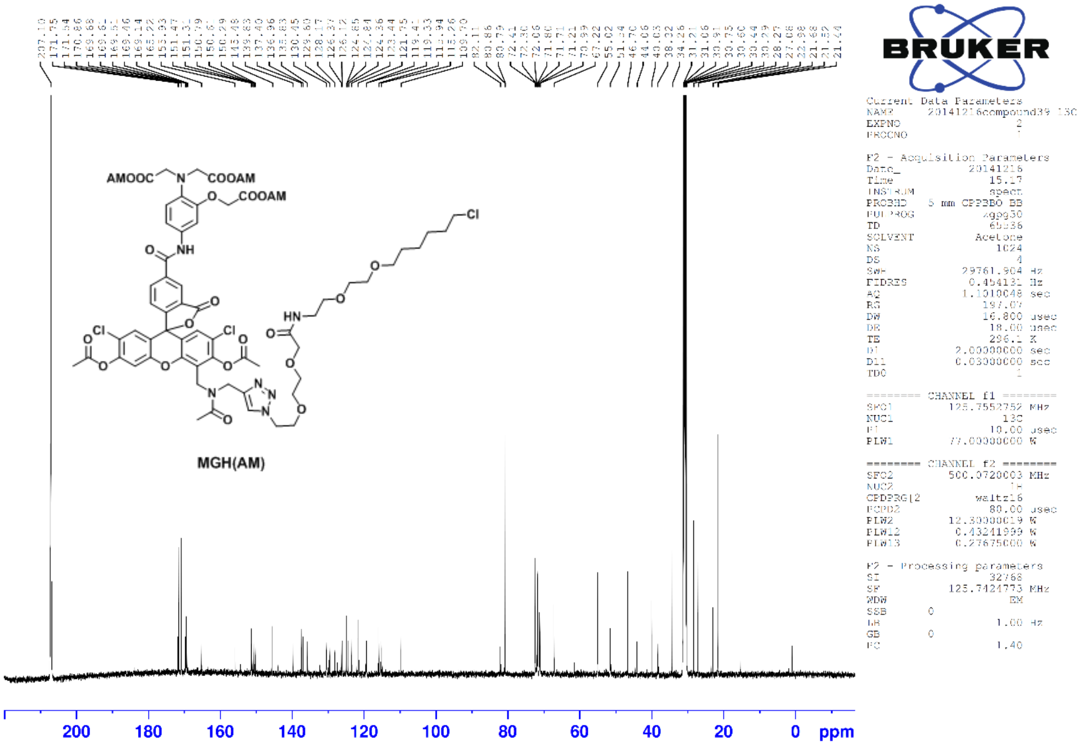

^{13}C NMR spectrum of MGH(AM)

3. Supplementary References

(S1) S. Watanabe, S. Mizukami, Y. Akimoto, Y. Hori and K. Kikuchi, *Chem. Eur. J.*, 2011, **17**, 8342–8349.

(S2) Y. Hori S. Hirayama, M. Sato and K. Kikuchi, *Angew. Chem. Int. Ed.*, 2015, **54**, 14368–14371.

(S3) B. Metten, M. Smet, N. Boens and W. Dehaen, *Synthesis*, 2005, **11**, 1838–1844.

(S4) C. C. Woodroofe, R. Masalha, K. R. Barnes, C. J. Frederickson and S. J. Lippard, *Chem. Biol.*, 2004, **11**, 1659–1666.

(S5) X. Chen, S. McRae, S. Parelkar and T. Emrick, *Bioconjugate Chem.*, 2009, **20**, 2331–2341.
